# Pushing the limits of single-cell proteomics to investigate bacterial heterogeneity using bacSCP

**DOI:** 10.1038/s41467-026-77426-y

**Published:** 2026-09-01

**Authors:** Julia Leodolter, Tim Thierer, Karl Mechtler, Manuel Matzinger

**Affiliations:** 1 Research Institute of Molecular Pathology (IMP), Vienna BioCenter (VBC), Vienna, Austria; 2 Gregor Mendel Institute of Molecular Plant Biology (GMI), Austrian Academy of Sciences, Vienna BioCenter (VBC), Vienna, Austria; 3 Institute of Molecular Biotechnology (IMBA), Austrian Academy of Sciences, Vienna BioCenter (VBC), Vienna, Austria

**Keywords:** Mass spectrometry, Proteomic analysis, Bacteria, Microbiology techniques, Cellular microbiology

## Abstract

Single-cell proteomics (SCP) has emerged as a powerful approach to quantify protein expression variability at cellular resolution, yet most state-of-the-art workflows are tailored to eukaryotic cells with only one study exploring how single bacteria can be analyzed by mass spectrometry. Here, we established bacSCP, a protocol extending SCP to bacterial cells, facing analytical challenges such as the thick bacterial cell wall hampering lysis, the extremely small cell size and resultant low protein content, and the comparatively high level of contaminating proteins from external sources. Using this bacSCP pipeline, we quantified more than 50 bacterial proteins from single *Bacillus subtilis* and *Escherichia coli* cells. Upon heat stress, we reproducibly observed up to 8-fold upregulation of chaperones including GroEL, GroES, and ClpC for a *B. subtilis ΔmcsB* strain. Importantly, single-cell measurements revealed potential heterogeneity within the heat-stressed subpopulation, enabling interrogation of stress-response variability at the proteome level. These results demonstrate the feasibility of bacSCP and provide a foundation for studying bacterial stress adaptation and phenotypic diversity with single-cell proteomic resolution.

## Abstract (web summary)

Single-cell proteomics (SCP) can be used to quantify protein expression variability at cellular resolution. Here, Leodolter et al. develop an SCP method to quantify proteins in individual bacterial cells, revealing stress-related protein changes and heterogeneity within bacterial subpopulations.

## Introduction

Single cell proteomics (SCP) by mass spectrometry (MS) is an emerging technology that continuously pushes its limits towards improved reproducibility, throughput and sensitivity^1–3^. As the protein content of a single eukaryotic cell is very low in the 200 pg range, SCP requires state-of-the-art equipment and workflows to reduce sample loss in an extremely low input setting. Successful approaches apply semi-automated sample preparation within minimal volumes as was shown using the cellenONE^4–9^, which performs ultra-precise image-based cell sorting and droplet dispersion, using fluorescence-activated cell sorting (FACS)^10,11^, thermal inkjet dispensing (TECAN Uno)^12^, or laser capture microdissection for single-cell analysis with spatial resolution^13^. Downstream processing of cells within minimal volumes was shown to enhance efficiency and recovery, as initiated by Zhu et al. using nanoPOTS^8^. In line with this, a One-Pot reaction setup was shown to minimize losses^2,14,15^. The combination with ion mobility filters such as FAIMS was further reported to improve achievable sensitivity for single-cell proteomics by filtering out singly charged ions^16^, and recently we demonstrated that tuning FAIMS electrode temperatures can further enhance signal intensity and quantitative accuracy^17^. Using such lossless workflows and the Astral mass analyzer achieved remarkable coverage of more than 5000 proteins quantified on average per single cell was achieved, and measured abundances were confirmed using ground truth datasets^5,18^. Such workflows were applied to investigate heterogeneity based on different cell-cycle phases even from cultured untreated cells, and to precisely follow cell differentiation and map it to RNAseq data from large cohorts of thousands of cells^5,6,19^.

While eukaryotic SCP is already a challenging task necessitating the most advanced hardware, downscaling this technique to bacteria pushes the boundaries of current technology. With an expected volume of ~2 fL^20^, bacteria are approximately 1000 times smaller than eukaryotic cells ( ~ 2 pL)^21^. and thus contain much less protein, with bacterial cell content estimated to be below 1 pg protein even for actively growing cells^22^.

Major challenges with bacteria include efficient cell sorting due to small cell size and sample loss during handling due to low input material. Additionally, cell lysis needs to be compatible with downstream MS applications while effectively breaking the bacterial cell wall. Gram-positive bacteria (e.g., *Bacillus subtilis*) are surrounded by a thick peptidoglycan cell wall, while Gram-negative bacteria (e.g., *Escherichia coli*) have a peptidoglycan wall, a periplasmic compartment, and an outer membrane. To the best of the authors’ knowledge, there is currently only a single publication that successfully attempted first steps towards single bacteria proteomics, reporting 12 confident proteins from single bacteria using a modified SCoPE MS approach, where they multiplexed 9 cells plus an additional 250 cell carrier to push sensitivity^23^. Although this sensitivity boost was necessary with the hardware available at the time of publication, such DDA-TMT-based approaches suffer from a bias towards quantifying peptides present in the carrier proteome, which hampers potential detection of new or rare cellular subpopulations, as well as from limited dynamic range and ratio compression^2^.

Expanding on these results and establishing robust bacterial SCP will be critical to enabling direct proteomic studies of bacterial heterogeneity at the single-cell level. Phenotypic heterogeneity in isoclonal bacterial populations exists as a bet-hedging strategy, where individual bacteria stochastically shift to different metabolic states, allowing rapid adaptation to environmental changes, such as nutrient and temperature stresses^24,25^. Importantly, bacterial population heterogeneity plays a crucial role in persister cell formation during antibiotic stress and during infection^26^.

Analysis of bacterial heterogeneity at the single-cell level is already well established using fluorescence-based phenotypic observations of cellular processes, as well as through transcriptomics and metabolomics^25,27^. Integrating SCP with these existing approaches would provide a critical layer of information for understanding bacterial heterogeneity, as cellular regulation occurs not only on DNA and RNA, but also at the protein level, through post-translational modifications, targeted degradation and sequestration of key factors, especially during cellular stress^28,29^.

In bacteria, heat stress is a well-studied environmental challenge. During heat stress, the protein quality control machinery is upregulated and critical to maintain protein homeostasis and to protect cells from proteotoxic stress. The universal essential folding chaperonin GroEL-GroES refolds damaged proteins, while proteases such as the ClpCP system degrade damaged proteins^30^.

In this study, we establish the first label-free proof-of-concept approach for bacterial single-cell proteomics (bacSCP). Here, we adapt and expand existing methods developed for eukaryotic and bacterial SCP. We benchmark multiple approaches for bacterial cell isolation and lysis to enable efficient protein extraction and digestion. Applying this workflow to heat-stressed *B. subtilis ΔmcsB*, we detect upregulation of the critical heat shock response proteins ClpC, GroES, and GroEL. Furthermore, we assess whether heat-stressed and non-stressed cells can be distinguished at single-cell resolution and evaluate the extent of proteomic heterogeneity among individual bacteria.

## Results

### Sample preparation and workflow optimization

In this study, we investigate how to push the sensitivity of SCP to a level where single bacteria can yield meaningful results. We thereby combined and adopted our robust and sensitive One-Pot^4^ SCP sample preparation workflow with the capability to isolate bacterial cells, making use of the microLIFE mode on the cellenONE, and the enhanced sensitivity of our LC-MS platform^5^ by employing columns with integrated emitter tip and zero dead volume, short 12.5 min active gradients achieving a throughput of 80 samples per day (SPD), and the Orbitrap Astral MS. Our optimization process included screening gram-negative (*E. coli)* and gram-positive (*B. subtilis*) bacteria as well as staining and cell lysis conditions, including cell wall-deficient bacteria.

We started to tune sample preparation parameters for bacSCP using *E. coli* cells. We first aimed to directly apply our One-Pot workflow^4^, where cell lysis is enabled by the MS-compatible detergent N-Dodecyl-β-D-maltoside (DDM) and incubation at 50 °C only, to bacterial cells. To do so, we removed the peptidoglycan cell wall prior to sorting to facilitate bacterial lysis by DDM. Bacteria were treated with Lysozyme to generate protoplasts or spheroplasts surrounded by a cell membrane only. The resulting cell-wall-deficient bacteria are spherical-shaped and of <2 µm diameter as estimated on the cellenONE. While the success of cell wall digestion for selected cells can be ensured by their altered shape on the cellenONE robot, their accurate isolation proved challenging as cells were often detected outside the PDC capillary by the image detection software of the cellenONE. This was likely caused by minimal movements of the PDC, and dust or dirt particles by accident were detected as a cell. Activation of the reduce-shaking option on the cellenONE improved performance but did not yield satisfactory results. Manual validation of detected particles inside the capillary proved difficult since no clear cell shape was visible to the naked eye in the recorded pictures. As a result, alternative cell isolation and lysis procedures were explored.

**Fig. 1:**
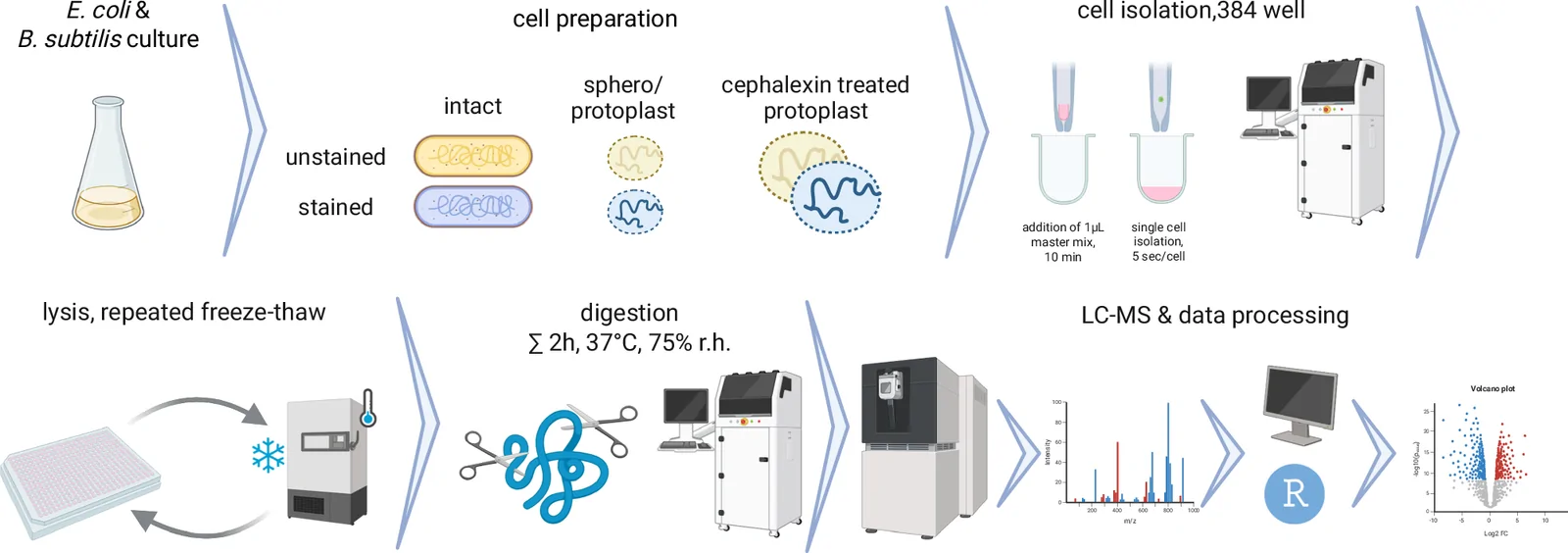
Workflow overview. To compare starting conditions, wild-type (intact) bacteria (stained or unstained) or cephalexin-enlarged protoplasts or spheroplasts were harvested at an OD600 of 0.7–0.9 followed by isolation of individual cells using the cellenONE robot. Cells were isolated into 1 µL of a pre-dispensed lysis and digestion mix within a 384-well plate. For intact cells, lysis was achieved by repeated freeze-thaw cycles. After stepwise digestion and a total of 2 h incubation, digestion was stopped by acidification using TFA, and cells were directly subjected to LC-MS analysis in the very same 384-well plate using an 8 cm IonOpticks Gen4 column at 80 samples per day (SPD) throughput and an Orbitrap Astral MS. Created in BioRender, Matzinger, M. (2026) (https://BioRender.com/2h63axl). The R logo was obtained from R^58^.

We subsequently investigated whether repeated freeze-thaw cycles followed by heating of intact bacteria lead to successful lysis as previously demonstrated by Végvári et al.^23^ Furthermore, we tested the effect of inhibition of cell division using cephalexin prior to cell wall removal, resulting in enlarged spheroplast cells^31^ and improved detectability on the cellenONE (estimated diameter by cellenONE 6.7 + /− 1.6 µm). Of note, we expect reduced physiological relevance for cephalexin-enlarged cells, but we decided to include this condition to explore the size limits needed for accurate detection using our setup on the CellenONE robot. Both strategies enabled us to successfully sort cells into individual wells of a 384-well plate. Introduction of a staining step (HOECHST 33342) ahead of sorting, with the resulting fluorescence signal used as additional selection criteria on the cellenONE, allowed us to improve the rate of confident successful isolations close to 100%. An overview of those tested workflow variations is depicted in Fig. 1.

**Fig. 2:**
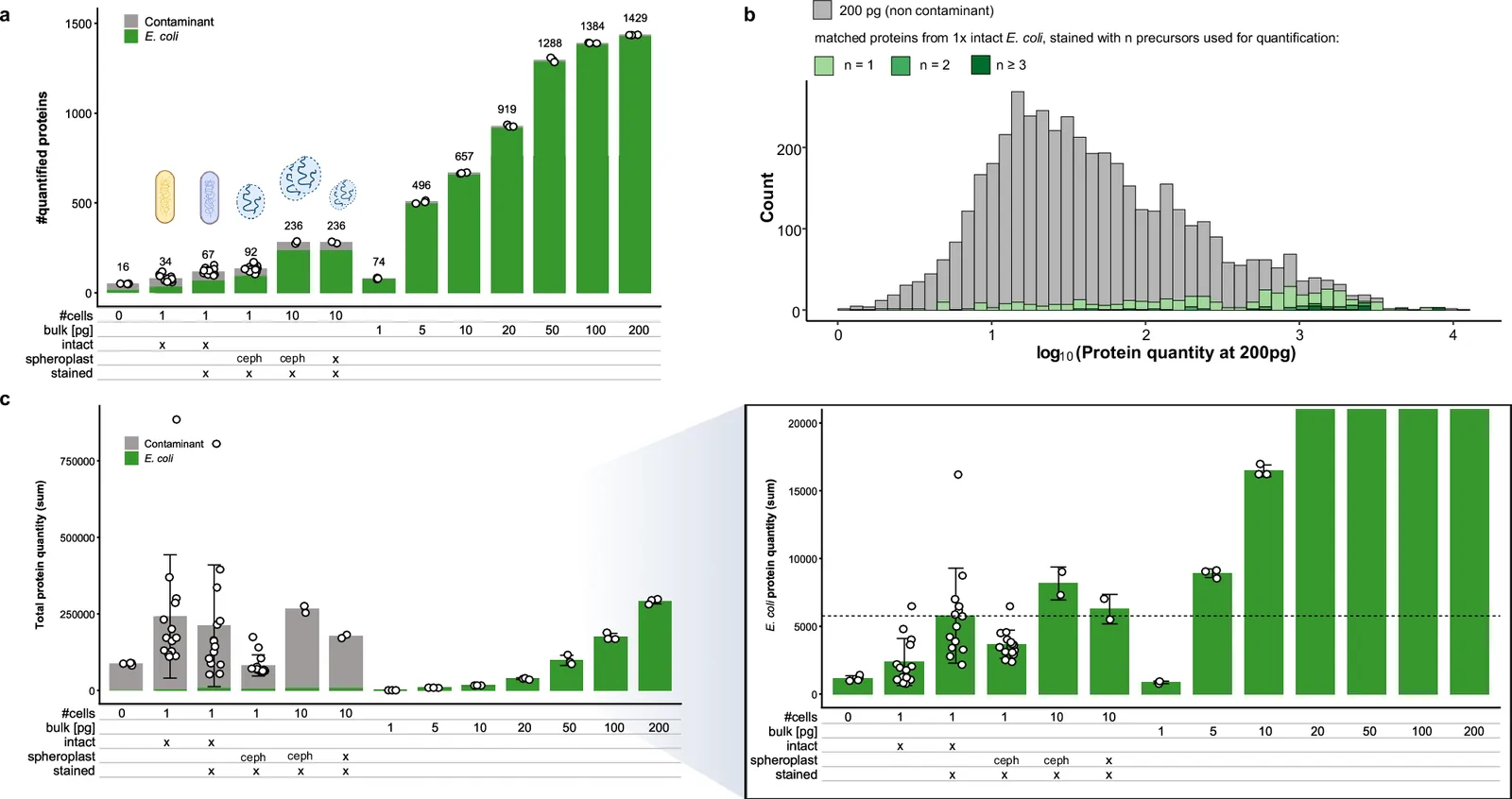
Establishing BacSCP using E.coli. ** a** Bars indicate the average number of quantified protein groups per single cell, spheroplast, 10 cells or from diluted bulks (injected at *n* = 3 replicates) as indicated. Cells indicated as ceph are treated with cephalexin. White circles indicate the number of quantified protein groups from individual replicates. Numbers above bars indicate the number of quantified non-contaminant proteins. **b** Histogram showing the distribution of protein quantities found from 200 pg diluted bulk and their matched quantities from all *n* = 14 measured single cell replicates of stained intact E. coli, with the indicated number of precursors used for protein quantification. **c** Bars show the average summed protein quantity per cell, ten cells or diluted bulks as indicated (left), as well as a zoom-in with quantities from contaminant proteins excluded (right). Error bars show their standard deviation.

We first compared the above-mentioned cell-isolation and lysis conditions using *E. coli* cells and found 34 to 67 quantified *E. coli* protein groups on average per cell from intact unstained or stained cells, respectively. The additional enlargement of spheroplasts using cephalexin yielded 92 quantified *E. coli* protein groups per cell on average, given that these spheroplasts originate from a fusion of several division-inhibited *E. coli* cells. On a limited number of replicates, we further analyzed 10 cell mini-bulks yielding 236 bacterial proteins on average, but surprisingly, no detectable effect on the number of quantified protein groups was originating from cephalexin treatment (Fig. 2a). We included the CRAPome^32^ database to account for contaminants, mainly originating from added trypsin as well as human skin keratins within our samples. Excluding those contaminants, we plotted the quantitative distribution of all proteins seen in 200 pg diluted *E. coli* bulk digests and matched the proteins found in single intact stained *E. coli* to the quantities from the bulk analysis. Consistent with our expectations, single-cell quantification within this distribution was biased toward high-abundance proteins (Fig. 2b). Of note, our 200 pg reference bulk already correlates to a low-input proteomic sample with input amounts comparable to single mammalian cells (i.e., HeLa cells). Further investigating the quality of quantified protein groups, we could show that more abundant proteins showed higher quality metrics, as seen for the correlation of the c-score (the discriminant score for Spectronaut to filter for a specific FDR, here 1%), the number of precursors per protein used for quantification, and data completeness to protein quantity, while the q value was unaffected (Supplementary Fig. 1). In line with this, when a filter of a minimum of 3 precursors used for protein quantification is applied, no bacterial proteins quantified remain within the 0 cell negative controls, which is to be expected as they are low abundant and by experimental design false positive hits (Supplementary Fig. 2). As above, filtering for a more stringent q value had no impact on the number of bacterial protein groups quantified in the negative control or their overlap with single cell samples (Supplementary Fig. 2). Within the fraction of quantified protein groups overlapping between negative control and single cell measurements, we selected a representative high-confidence high-abundance precursor of trypsin as well as a low-abundant but still confidently identified precursor of glycerinaldehyd-3-phosphat-dehydrogenase A and compared their extracted ion chromatograms. In both cases, high and low abundant precursor peak quality seems sufficient for proper integration and hence quantification (Supplementary Fig. 3).

To estimate the relative total abundance of all quantified protein groups in single cells or from 10 cells we compared the summed protein quantity to what was found from a titration of diluted bulk digests ranging from 1 pg to 200 pg (Fig. 2c). Consistent with previous reports^23^ and our expectations, up to 98% of the total protein quantity detected in single cells originated from contaminants, reflecting both the low protein content of individual bacteria and the necessary large excess of trypsin added per sample. Excluding those contaminants allows for improved comparison to the mini-bulks and an estimated 1–5 pg protein content extracted from single bacteria, or ~5 pg from 10 cells, respectively. Assuming that the 10-cell mini-bulk provides more accurate estimates, we infer a protein content of <1 pg protein per *E. coli* cell. This estimate is consistent with the value reported by Lenski et al.^22^, who reported 865 fg protein per *E. coli* cell when in late exponential phase, which matches the growth state of our cells.

To analyze the distribution of molecular processes associated with our single cell protein hits compared to bulk data, we mapped gene ontology (GO) slim terms to the respective protein hits (Supplementary Fig. 4a). In bulk data, proteins involved in primary metabolic processes constitute the major fraction of annotated proteins, while in single cell protein hits this is shifted in favor of proteins belonging to the translation group reflecting the high abundance of ribosomes in the cell^33^ and the fact that we detect predominantly highly abundant proteins from single bacteria (Fig. 2b). For stained single cells (intact or cephalexin-enlarged spheroplasts) we detect a higher amount of GO biological process groups, likely due to a higher number of absolute protein hits (Fig. 2a). Mapping the GO cellular compartments (Supplementary Fig. 4b), we observe a slight overrepresentation in plasma membrane associated proteins in the cephalexin-treated sample, potentially reflecting a higher membrane fraction or dysregulation of membrane components due to antibiotic treatment.

**Fig. 3:**
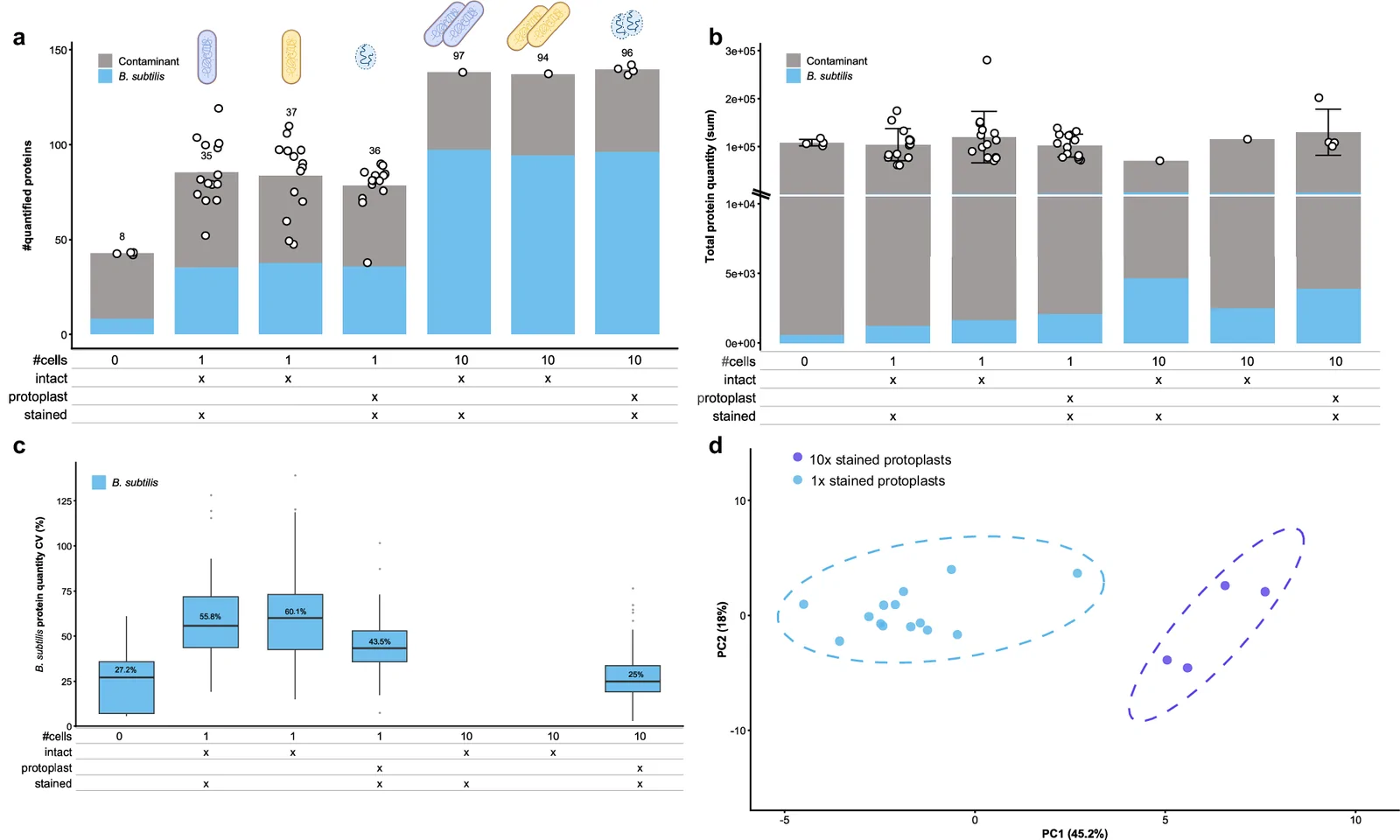
Setup of BacSCP using B. subtilis wild-type. Bars indicate the average number of quantified protein groups (**a**) or the average summed protein quantity (**b**) per single cell, protoplast or 10 cells as indicated. White circles indicate results from individual replicates. Numbers above bars indicate the number of quantified non-contaminant proteins. Error bars show standard deviations. Bacteria images were created in BioRender, Matzinger, M. (2026) (https://BioRender.com/2h63axl). **c** The Boxplot shows the coefficient of variation of protein group quantities for all non-contaminant proteins quantified in at least *n* = 3 out of *n* = 4, 14 and 10 replicates measured for 0 cell negative controls, single cells and 10x protoplast minibulks respectively. Intact 10x mini-bulks were measured only once which is why no CV can be calculated there. The boxplots show the median (center line), 25th and 75th percentile (box bounds), and whiskers extending to the largest/smallest value within 1.5x the interquartile range from the hinges. **d** Principal Component Analysis from all *n* = 14 measured stained single protoplasts and *n* = 4 mini-bulks from stained 10x protoplasts.

Based on the success of isolation as confirmed by fluorescence and the aim to remain as close as possible to native conditions prior to lysis, we selected intact stained cells as the preferred condition. We investigated the performance of our bacSCP workflow using gram-positive *B. subtilis* bacteria, applying the most promising conditions identified from the previously recorded *E. coli* data. Specifically, we focused on intact cells and protoplasts to assess potential effects on lysis efficiency. We quantified ~36 bacterial proteins on average per cell and ~95 proteins per ten cells independent of the sample preparation strategy (Fig. 3a) and a comparable ratio of bacterial to contaminant-originating protein quantity as seen for *E. coli* (Fig. 3b).

Coefficients of variation (CV) from bacterial protein quantities rise to ~60% on a single-cell level and 25% for 10 cells, respectively (Fig. 3c). Although this value appears high at first sight, it is consistent with expectations as single-cell measurements using fluorescent protein reporters have revealed substantial cell-to-cell variability in protein expression, with a CV of ~0.4^34^. While technical variability for 250 pg inputs was below 10% using our setup^5^, we expect that at substantially lowered protein inputs, as encountered on the single bacterium level, technical variability can increase to 20% or higher, as observed for the 10 cell samples in Fig. 3c. Combined with biological variability, this results in an overall CV of ~60%. A higher technical noise level for the majority of quantified precursors and proteins is to be expected based on a median of only 2 datapoints sampled by the MS per elution peak per precursor and only 2 precursors on median used for quantification per protein (Supplementary Fig. 5). Of note, some precursors yielded much higher numbers of up to 20 datapoints per elution peak, likely yielding an improved quality of quantification. We noted that the CV for the zero-cell negative control seems lower than that of single cells, although no bacterial input is present. We hypothesize that the signal in the negative control is dominated by noise rather than signals from true peptides. This observation might be explained by the narrower Gaussian distribution of noise levels due to the lack of biological variation. When including contaminants into the CV calculation, which introduces real peptide signals to that sample condition, the median CV drops marginally (from 27 to 25%, Supplementary Fig. 5c). Filtering for quantified protein groups based on at least three precursors removes all hits of the zero-cell negative control, confirming no confidently quantified bacterial protein hits hidden in the negative control (Supplementary Fig. 5d). Applying the same filter helped to reduce the median CV of stained intact single cell samples from ~56% down to 32%, presumably improving quantification accuracy.

Principal Component Analysis (PCA) of single cells versus 10 cells shows a clear separation of sample groups (Fig. 3d), proving that quantitative differentiation of single to multiple cells is sufficient using a label-free workflow. Together with the differences in summed protein quantities shown in Fig. 3b, this finding corroborates earlier results reported by Végvári et al.^23^ who employed an isobaric multiplexed strategy with a carrier proteome to enhance sensitivity on an Orbitrap Eclipse MS in 2023.

### Estimation of required sensitivity level for identifying biologically relevant protein regulation

After establishing the bacSCP workflow, we next aimed to validate whether biologically relevant protein regulation can be detected at the single-bacterium level. To this end, we used heat stress to investigate how much protein input could be reduced while still detecting the upregulation of the critical heat stress chaperones GroEL-GroES and ClpC. In *B. subtilis*, the ClpCP complex degrades proteins marked by the unusual post-translational modification phospho-arginine (pArg), and pArg-dependent degradation is critical for survival after prolonged heat shock^35^. Phosphorylation of protein arginine residues is performed by the protein arginine kinase McsB and counteracted by the pArg phosphatase YwlE^36–38^. Here, we selected a *B. subtilis ΔywlE* strain, which is deficient in the pArg phosphatase YwlE and an established strain for detecting pArg-dependent effects^37,39^.

**Fig. 4:**
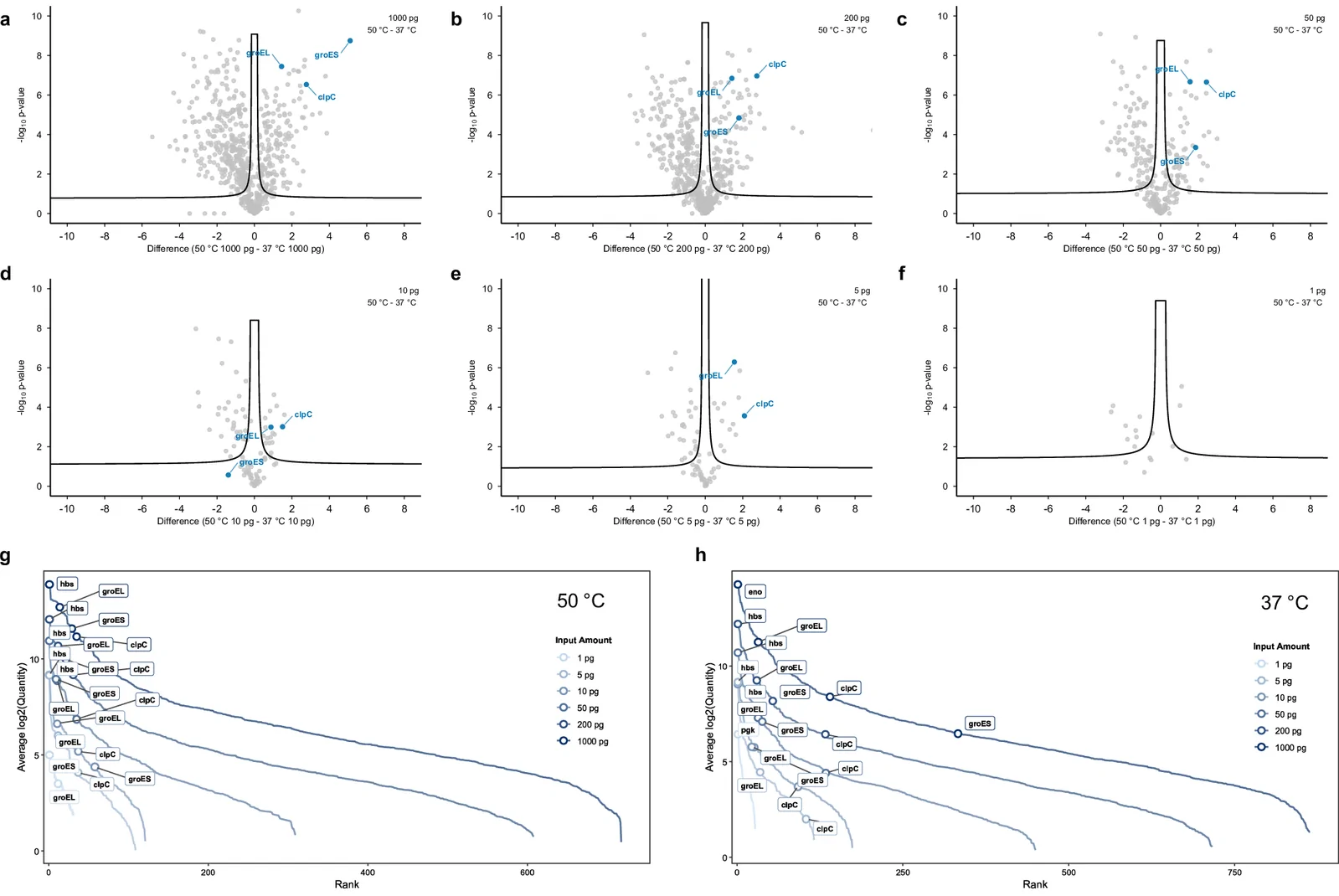
Sensitivity titration using B. subtilis ΔywlE bulk samples. **a–f** Volcano plots from 1 to 1000 pg diluted bulk samples from heat shocked (50 °C) vs non heat shocked (37 °C) B. subtilis. groEL, groES and clpC are highlighted. *n* = 3 technical replicates, no imputation and normalization applied. *P*-values were calculated using a two-sided Student’s t-test with permutation-based multiple-testing correction (FDR < 0.05); see Methods for details. **g**,**h**. Ranked quantified protein groups by their average quantity from those *n* = 3 replicates for heat shocked (left) or non-heat shocked (right) samples, with the most abundant protein and proteins as in (**a–f**) highlighted.

To assess if the sensitivity given by our existing LC-MS setting is sufficient to accurately quantify relevant heat shock proteins, we first acquired data from diluted bulk samples ranging from 1 to 1000 pg total input. We find that the regulation of GroEL, GroES, and ClpC upon heat stress is visible and remains significant for inputs down to 50 pg (Fig. 4a–c). For very low inputs of 10 pg and less, data quality starts to decline (Fig. 4d–f) and becomes sparser. At 1 pg injection, these proteins were below their limit of quantification at 37 °C, while upon heat stress, GroES and GroEL are quantified as evident in the ranked proteins graphs (Fig. 4g, h).

### Heat shock response in *B. subtilis* wild type and mutants

**Fig. 5:**
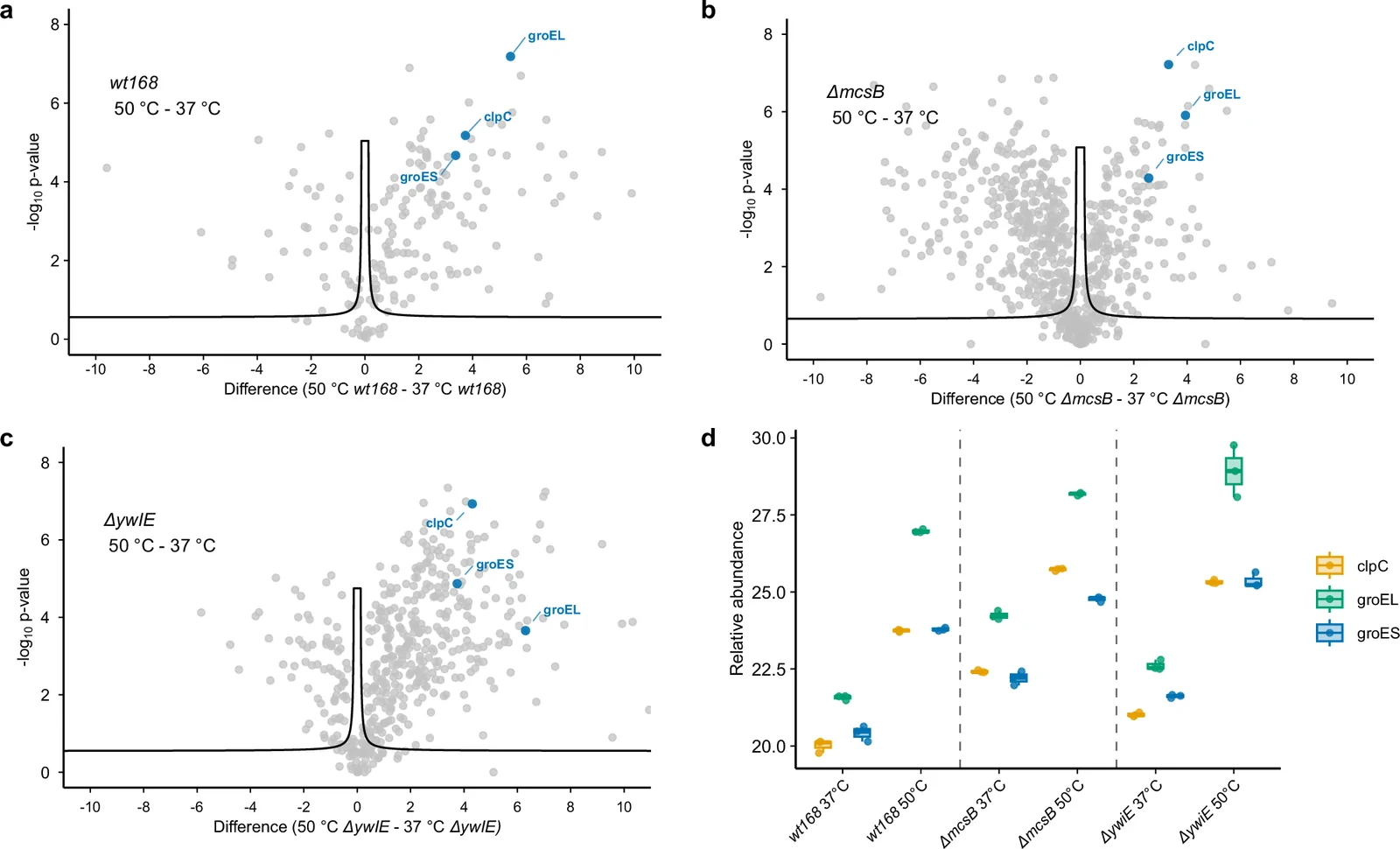
Comparison of B. subtilis variants. Volcano plots from 200 ng bulk samples from heat shocked (50 °C) vs non heat shocked (37 °C) wt168 (**a**), ΔmcsB (**b**) or ΔywlE (**c**) B. subtilis with relevant chaperons expected to be upregulated upon heat-shock highlighted. *n* = 3 technical replicates, no imputation and normalization applied. *P*-values were calculated using a two-sided Student’s *t*test with permutation-based multiple-testing correction (FDR < 0.05); see Methods for details. **d** The boxplot indicates the distribution of relative protein abundance of those highlighted proteins after label-free quantification. The boxplots show the median (center line), 25 and 75th percentiles (box bounds), and whiskers extending to the largest/smallest value within 1.5x the interquartile range from the hinges. *n* = 3 technical replicates.

Given the evident sensitivity of the bacSCP workflow to successfully quantify heat-shock chaperones, we next benchmarked this regulatory response across different *B. subtilis* mutant strains, aiming to select the best cell line for a later single-cell level experiment. We tested *B. subtilis ΔywlE* and *ΔmcsB*, and the wild-type strain (Fig. 5). Both *ΔywlE* and *ΔmcsB* are disrupted in pArg-dependent regulation, as either the pArg phosphatase YwlE or the pArg kinase McsB is knocked out, resulting in substantially increased pArg levels or complete lack thereof. ClpC as well as GroEL were shown previously to be modified by pArg and upregulated upon YwlE knockout^37,39^. Here, ClpC, GroEL and GroES are significantly upregulated during heat shock in all conditions tested (Fig. 5). While in *B. subtilis ΔmcsB* the fold change of upregulation is lower than for wild-type and *ΔywlE* (Fig. 5d), ClpC ranks among the top 10 most abundant proteins, prioritizing this strain for future heat-stress experiments.

### BacSCP enables sensitive and robust detection of the heat-shock response at the single-bacterium level

**Fig. 6:**
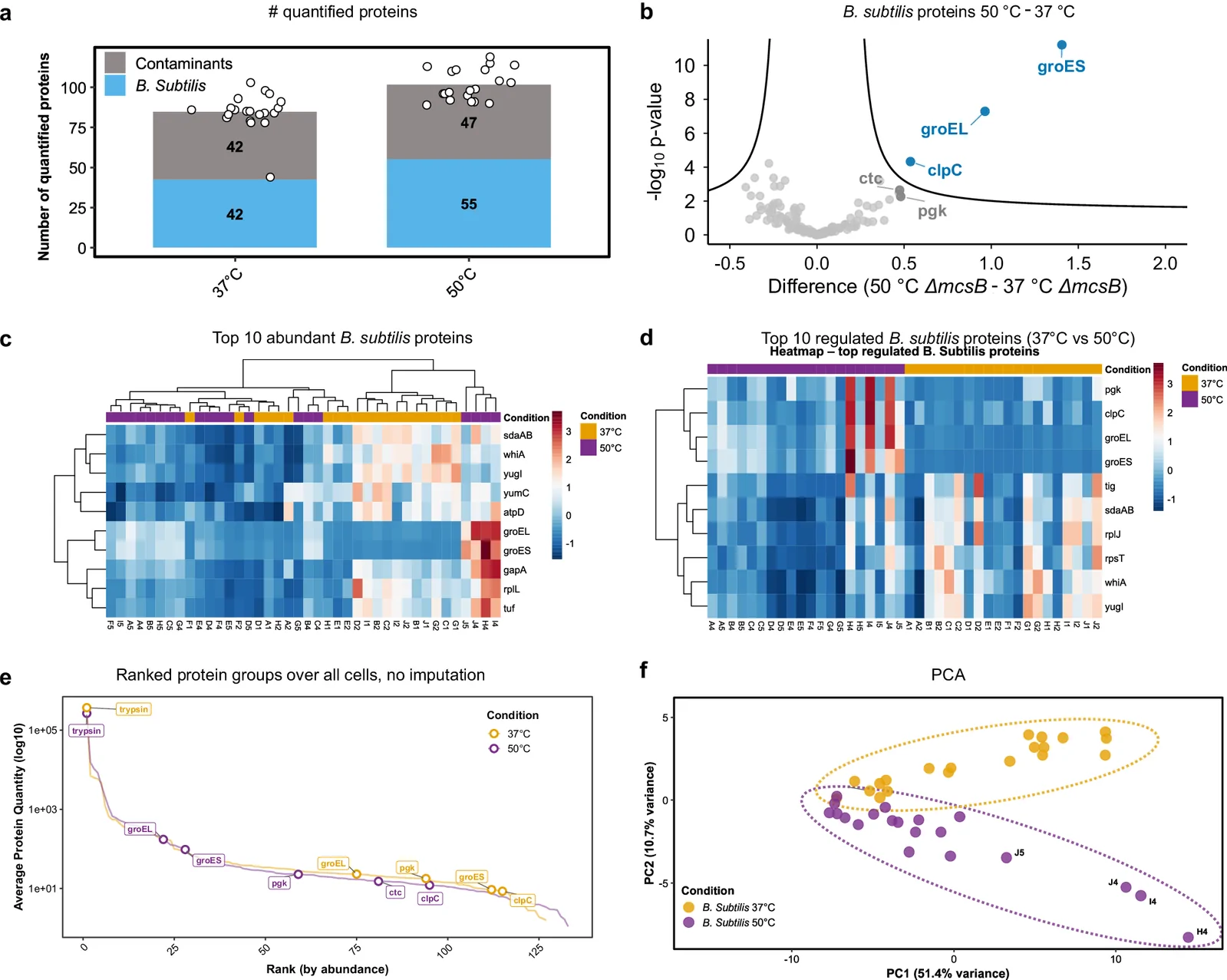
Investigation of heat shock response on single B. subtilis *ΔmcsB* cells. **a** Bars indicate the average number of quantified protein groups from a single B. subtilis with (50 °C) or without (37 °C) heat-shock, *n* = 20 individual cells per condition. **b** Volcano plot showing regulated non-contaminant quantified protein groups upon heat-shock. **c**,**d** Heatmaps showing relative quantities of the top 10 most abundant or top 10 upon heat-shock regulated proteins. *P*-values were calculated using a two-sided Student’s t-test with permutation-based multiple-testing correction (FDR < 0.05); see Methods for details. **e**. By quantity ranked protein groups found in all *n* = 20 single cells measured/condition with most regulated proteins as seen in B highlighted. **f**. Principal component analysis from heat-shocked (50 °C) and non-heat-shocked (37 °C) single B. subtilis measured.

Finally, we applied our optimized bacSCP workflow to assess whether the heat shock response can be reliably quantified at the single-bacterium level and whether cell-to-cell proteomic heterogeneity can be detected. For this proof of principle study, we selected *B. subtilis ΔmcsB* as this cell line showed the highest relative abundance of ClpC in our pilot experiment (Fig. 5). We analyzed 20 single bacteria for each condition, harvested at 37 °C or after a 50 °C heat shock treatment. Intact and stained cells were sorted, yielding 42 and 55 bacterial proteins groups quantified per cell on average for non-heat-shocked and heat-shocked cells respectively (Fig. 6a). In line with our observations in *E. coli*, those proteins predominantly represent abundant proteins found from reference bulk proteomic runs (Supplementary Fig. 6). All three investigated chaperones showed a significant upregulation when comparing average quantities from all *n* = 20 single cells measured per condition (Fig. 6b) and their upregulation also becomes obvious by their shift in a ranking of all quantified protein groups (Fig. 6e). Further, a slight effect was observed for Pgk and Ctc, with Ctc only quantified after heat-shock. The ribosomal protein Ctc is known as a general stress response protein that associates with ribosomes under stress^40^, while Pgk is an essential glycolytic phosphoglycerate kinase, and both proteins were found to be upregulated during heat stress in wild-type *B. subtilis*^41^.

To investigate potential heterogeneity within the analyzed cell population, we plotted a heat map of the top abundant and top regulated proteins (Fig. 6c, d). A list of these proteins including their biological function and reported pArg modification sites is shown in Table 1. As observed for *E. coli*, the major biological processes involved are translation and metabolic enzymes, now accompanied by protein quality control factors induced by heat shock. We found a well-defined subset of proteins mainly upregulated upon heat-shock, with GroEL and GroES among the strongest upregulated candidates. Protein levels of GroEL, GroES and ClpC are relatively uniform at 37 °C (more so for GroEL/ES than for ClpC) while protein levels after heat shock differ markedly. We observe three groups of single cells where the targeted proteins of interest are either barely, intermediately or highly upregulated, indicating strong heterogeneity in the response of individual cells to heat stress. Furthermore, a subset of 4 cells seems to show a much stronger heat-shock response and by that defines a separate cluster (Fig. 6c). Considering high technical run-to-run variability expected for such ultra-low input proteomic samples (see Fig. 3c), we suspect that the visible heterogeneity might at least partly result from technical variance and partly from biological heterogeneity. A significantly enlarged sample size would be required in future studies to improve statistical power and confirm this hypothesis. Given that this is the first study showing biological regulation at the single bacterium level, we believe our data provides valuable first insights towards cell-to-cell heterogeneity present within cultured bacteria. This pattern is further reflected in the PCA, which shows a well-defined separation of non-heat-shocked and heat-shocked cells. Cells exhibiting the strongest GroEL and GroES responses, together with other proteins highlighted in the heatmap, form a distinct population that is also clearly resolved in the PCA (Fig. 6f). In our *E.coli* based test dataset, we found that proteins quantified based on at least 3 precursors show higher quantitative confidence, and no non-contaminant proteins remain in 0 cell negative controls after applying this filter (see Supplementary Fig. 2). When we applied this filter to the heat-shocked *B. subtilis* dataset, we found that 9 (out of 42 with standard filter) and 13 (out of 55 with standard filter) bacterial protein groups were still quantified at high confidence (Supplementary Fig. 7a). Visible cell-to-cell heterogeneity and clustering remained largely unaltered, which is to be expected as these patterns were already primarily driven by the most abundant proteins prior to filtering (Supplementary Fig. 7c, d, f). Analysis of significantly regulated proteins again showed groES and clpC upregulated upon heat shock, while groEL loses its significance in the volcano plot, but nevertheless remains in the Top10 regulated proteins as shown by the heat map. Instead tpiA and dnaK surface as significantly regulated after filtering (Supplementary Fig. 7b, e). DnaK is a class I heat-shock chaperone of the Hsp70 family, and TpiA is a triose phosphate isomerase, a metabolic enzyme involved in glycolysis/gluconeogenesis, that is strongly upregulated in the *B. subtilis ΔmcsB* strain during heat stress ( ~ 6.5 fold increase in the bulk experiment in Fig. 5). Both proteins were reported among the 100 most abundant proteins in *B. subtilis*^42^. In any case, independent of the filter settings used, we detect upregulation of relevant protein quality control systems upon heat shock.

**Table 1:** Top10 upregulated and Top10 abundant proteins from Fig. 6C and D

Gene Name	Uniprot ID	Protein Name (UniProt)	Top 10 regulated	Top 10 abundant	Biological Process (GO)	pArg reported
*yumC*	O05268	Ferredoxin--NADP reductase 2		✓	detoxification	
*pgk*	P40924	Phosphoglycerate kinase	✓		primary metabolic process	
*atpD*	P37809	ATP synthase subunit beta		✓	primary metabolic process	
*gapA*	P09124	Glyceraldehyde-3-phosphate dehydrogenase 1		✓	primary metabolic process	✓
*sdaAB*	O34635	Probable L-serine dehydratase, beta chain	✓	✓	primary metabolic process	
*tig*	P80698	Trigger factor	✓		protein folding	✓
*groEL*	P28598	Chaperonin GroEL	✓	✓	protein folding	✓
*groES*	P28599	Co-chaperonin GroES	✓	✓	protein folding	
*clpC*	P37571	Negative regulator of genetic competence ClpC/MecB	✓		protein folding	✓
*tuf*	P33166	Elongation factor Tu		✓	translation	✓
*rplL*	P02394	Large ribosomal subunit protein bL12		✓	translation	
*rplJ*	P42923	Large ribosomal subunit protein uL10	✓		translation	
*rpsT*	P21477	Small ribosomal subunit protein bS20	✓		translation	
*yugI*	P80870	General stress protein 13	✓	✓	unannotated	
*whiA*	O06975	Probable cell division protein WhiA	✓	✓	unannotated	

Biological Process GO slim data was retrieved from the QuickGo^56^ database, modification by pArg from Schmidt et al.^39^ and Elsholz et al.^37^

## Discussion

We demonstrate that bacSCP is applicable to both *E. coli* and *B. subtilis*, representing Gram-negative and Gram-positive model organisms, enabling comparable depth of non-contaminant protein detection in *B. subtilis* and *E. coli* (~ 55 vs 65 respectively). In accordance with cellular abundance, we predominantly detect metabolic enzymes and ribosomes, and in the case of heat-stressed *B. subtilis*, stress response chaperones. However, our technique also detects proteins in the medium abundance range which are exclusively proteins quantified based on only one precursor (Fig. 2b, Supplementary Fig. 6). While identification of such proteins close to the limit of quantification seems to be still confident based on the quality metrics given by Spectronaut, their accurate quantification is currently unfeasible. Being able to identify them nevertheless highlights the sensitivity of our method. Of note, the only previous single bacterial cell study published in 2023^23^ used a multiplexed approach including a 250 cell carrier channel in order to boost sensitivity to a level where quantitative differentiation of single to multiple cells was possible. With current state of the art loss-less workflows and sensitive MS instrumentation, such a differentiation is possible by means of label free proteomics without the use of any library or carrier (Fig. 3d).

Strikingly, we detected the upregulation of GroEL, GroES and ClpC in heat-stressed *B. subtilis* samples at the single cell level. This effect was most pronounced in four individual cells (Fig. 6d). Interestingly, GroEL/ES and ClpC are under the transcriptional control of different repressors, HrcA and CtsR respectively^43,44^, but in cells we observe concurrent increased protein abundance of ClpC, GroEL/ES as well as the not stress related protein Pgk, indicating an overall heat stress effect rather than effects specific to certain proteins or regulons. However, caution is required when interpreting these results, due to the very low input sample and the high technical variability, some observed effects could be technical rather than biological in nature. To more confidently distinguish between biological and technical variance, future experiments that extend beyond a proof-of-concept study should consider a larger sample size.

Applying bacSCP to a broader range of bacterial species in future experiments would provide deeper insight into the generalizability and robustness of our workflow. Of particular interest is the application of this method to pathogenic bacteria which is of interest to provide valuable insights into both virulence and antibiotic susceptibility. The evident capacity of bacSCP to detect ClpC invites exciting possibilities for its application to *Staphylococcus aureus* where the ClpC operon via McsB is required for virulence^45^, and Mycobacteria (e.g. *Mycobacterium tuberculosis, Mycobacterium smegmatis*), where ClpC1 is a target of promising antimicrobial compounds like cyclomarin or ecumicin^46,47^.

To further investigate heterogeneity in pathogenic bacteria and to resolve proteins involved in broader antibiotic tolerance and resistance, bacSCP will require further improvements in sensitivity, as pathologically relevant proteins are often not among the most abundant proteins in the cell^48^. Nevertheless, we anticipate that ongoing technical and methodological advances will continue to push the detection limits of bacSCP. While single-bacterial-cell analysis has been established using other approaches, including phenotypic characterization with fluorescent reporters, single-cell transcriptomics, and metabolic profiling by mass spectrometry^25,27^, this study represents, to our knowledge, the first demonstration of bacterial single-cell proteomics capable of detecting stress-induced proteomic changes at the level of individual cells.

## Methods

### Culturing bacterial cells

*B. subtilis ΔywlE* was generated in Schmidt et al.^39^ and *B. subtilis ΔmcsB* was generated in Suskiewicz et al.^49^ *Escherichia coli* strain BL21(DE3), *B. subtilis* strain 168, wild-type and *ΔywlE* and *ΔmcsB* knockout strains were inoculated from a glycerol stock into LB medium and grown overnight at 37 °C with constant agitation of 140–160 rpm. The main culture was inoculated with 1/100 of the overnight culture. At an OD_600_ of 0.7–0.9 samples were either harvested by centrifugation at 4 °C, or transferred to a preheated shaker and subjected to heat shock for 20 min at 50 °C and subsequently cooled down on ice and harvested. Cell pellets were washed 3x times with PBS, resuspended in Buffer 50 mM Hepes pH 7.3, 100 mM NaCl + 25% glycerol, flash frozen in liquid N_2_ and stored at −80 °C until analysis. Stained cells were incubated before analysis in a 1:1000 Hoechst 33342 (Thermo Scientific Pierce) dilution in PBS for 30 minutes at room temperature in the dark and washed 3x in PBS and diluted to ~200 cells/µL prior to sample analysis.

### Generation of protoplast/spheroplast cells

Wild-type and cephalexin-enlarged *E. coli* spheroplasts were prepared as described in Figueroa et al.^31^ In short, *E. coli* were grown as described in 25 mL LB to an OD_600_ of 0.7-0.9 and intact *E. coli* samples were harvested. To produce cephalexin-treated *E. coli* cells were diluted 1:10 in 30 mL LB containing 60 µg/mL cephalexin (Sigma Aldrich) and incubated at 37 °C for 2.5 h. Filaments were harvested at 4 °C. To produce spheroplasts, cell pellets were washed with 0.8 M sucrose and incubated in 350 mM Tris-Cl pH 7.8, 1.5 mg/mL Lysozyme, 0.35 mg/mL DNAse and 35 mM EDTA pH 8 for 10 min at room temperature. 1 mL solution A (20 mM MgCl2, 0.7 M sucrose, 10 mM Tris-Cl, pH 7.8) was added and cells incubated for 4 min. Subsequently, 7 mL of solution B (10 mM MgCl2, 0.8 M sucrose, 10 mM Tris-Cl, pH 7.8) were added at 4 °C, and pellets gently harvested and resuspended in 700 µL Solution B.

*B. subtilis* protoplasts were prepared similar to Chang & Cohen^50^. *B. subtilis* were grown as described in 25 mL LB to and OD_600_ of 0.7–0.9 and harvested. Pellets were resuspended in 4 mL of SMM (0.5 M sucrose, 0.02 M maleic acid pH 6.5, 0.02 M MgCl2) supplemented with 2.5 mg/mL Lysozyme and incubated for 35 min at 37 °C under gentle agitation. Pellets were harvested and resuspended in 500 µL SMM. Success of cephalexin treatment and spheroplast/protoplast formation was confirmed by bright field microscopy. Aliquots of the suspension were flash frozen in liquid N_2_ and stored at -80 °C until analysis.

### Sample preparation for *B. subtilis* bulk samples

For bulk analysis, pellets of the respective *B. subtilis* strains were thawed in the presence of Benzonase endonuclease and 5 mM MgCl2 and lysed by bead beating (Precellys 24, Bertin Technologies). 1.5 mL screw cap Eppendorf tubes were filled with ~500 µL of acid-washed glass beads <106 µm (Sigma Aldrich) and 700 µL of cell suspension and run for 6800 rpm, 3 × 20 s, 30 s pause at 4 °C. Lysates were cleared by centrifugation at 4 °C. Lysate concentration was measured by BCA Assay (Pierce). Lysates were diluted to 1 mg/mL and digested using trypsin (Trypsin Gold, V5280, Promega, 500 ng per sample) over night at 37 °C. Digestion was stopped by acidification to a final concentration of 1% (v/v) TFA and peptide concentration was estimated by means of UV-VIS chromatography at 214 nm using a monolithic (PepSwift™ Monolithic 50 × 0.2 mm) using the following gradient at a constant flow of 1.5 µL/min: 3 min 2% eluent B (80% ACN, 0.04% TFA), increasing to 95% over 20 min and kept at 95% for 5 min, decreasing to 2% in 1 min before re-equilibrating at 2% for 6 min. Eluent A was 0.05% TFA in water.

### Single cell proteomic sample preparation

Isolation of single intact *E. coli* or *B. subtilis* as well as their protoplasts/spheroplasts, cephalexin treated or stained cells was performed within the cellenONE X1 Neo robot. Thereby cells were sorted into a 384 well plate (Eppendorf twin-tec PCR LoBind) followed by lysis and digestion based on our previously published One-Pot protocol^4^ with the following details: Cells were sorted into wells containing 1 µL of master mix (0.2% DDM (D4641-500MG, Sigma-Aldrich), 100 mM triethylammonium bicarbonate (TEAB; 17902-500 ML, Fluka Analytical), 3 ng µL^–1^ trypsin (Trypsin Gold, V5280, Promega) and 1% DMSO). Humidity and temperature were controlled at 30% and 10 °C during cell sorting. Cells were isolated in microLIFE mode and stained cells were furthermore sorted in the basic cellenONE mode using the following parameters: Allowed size 4–10 µm (sizes starting at 1 µm for microLIFE mode) at a maximum elongation of 2, for stained cells with blue fluorescence channel active at 40% gain and 40 ms exposure time, selection with a minimum fluorescence intensity of 10. Protoplasts and spheroplasts were directly heated with lysis happening in parallel to digestion. For intact cells, lysis was facilitated by 5 freeze thaw cycles of at least 30 min freezing time at -70 °C and subsequent thawing at room temperature for 15 min each and protein digestion was performed at 50 °C and 80% relative humidity for 30 min before an additional 500 nL of 3 ng μL^–1^ trypsin was added followed by incubation for final 1.5 h at 37 °C and 70% relative humidity. Digestion was stopped by addition of 2 µL 0.5% (v/v) TFA and storage at −20 °C until MS analysis. For LC–MS/MS analysis, samples were directly injected from the 384-well plate.

### LC–MS analysis

Samples were analyzed using the Thermo Scientific Vanquish Neo UHPLC system. Peptides were separated on an Aurora Rapid 8×75 XT Gen4 nanoflow UHPLC column with an integrated emitter (IonOpticks) at 50 °C using a NanoShield C18 prototype trap column (IonOpticks).

To balance depth and throughput peptide separation was performed at 80 SPD based on previous experience^5,17^ and without any dedicated optimization for single bacteria samples with the following details: For the first 0.5 min a flow rate of 1 µL/min at 12% buffer B (80% ACN, 19.9% ddH2O, 0.1% TFA) was applied. Until minute 0.6 the flow rate was reduced to 250 nL/min at 13% buffer B followed by a stepwise gradient to 22% buffer B until minute 3, 37% buffer B until minute 10.5 and 56% buffer B until minute 12.5, respectively. After that the flow rate increased to 1 µL/min at 99% buffer B until minute 13 for column washing and was kept in this state for 1 min until minute 14. Fast sample loading was performed at a maximum pressure of 800 bar and at a maximum flow rate of 10 µL/min.

For MS measuring, the Orbitrap Astral MS (Thermo Scientific) equipped with a FAIMS Pro Duo interface (Thermo Scientific) and an EASY-Spray source was coupled to the LC. FAIMS was used in low resolution mode^17^ with an outer electrode temperature of 80 °C and an inner electrode temperature of 100 °C at a compensation voltage of –48 V. MS1 spectra were recorded using the Orbitrap analyzer at a resolution of 240,000 from m/z 400 to 800 using an automated gate control (AGC) target of 500% and a maximum injection time of 100 ms. For MS2 in DIA mode using the Astral analyzer, non-overlapping isolation windows of m/z 20 and a scan range from an m/z of 400 to 800 was chosen. A precursor accumulation time of 60 ms was allowed for and the AGC target was set to 800%.

### Data analysis

All raw data were analyzed using Spectronaut (version 20.4.260109.92449, Biognosys^51^). Data was searched library free using DirectDIA+ with replicates defined as the same condition in the search settings. Method evaluation mode was turned on to avoid transfer of IDs across conditions or to 0 cell negative control samples. Quantification was performed on MS2 level. Carbamidomethylation of cysteines as a static modification was removed as no alkylation step was performed with factory settings used besides that. Searches were performed against the *E. coli* K12 proteome (uniport reference proteome Tax ID 83333 as of 2025-06-30, 4402 sequences) or *B. subtilis* strain 168 proteome (uniport reference proteome, Tax ID 224308, as of 2025-06-27, 4194 sequences) respectively with the CRAPome^32^ (118 protein sequence entries) added to all searches as contaminant database.

### Post-processing

Statistical analysis and visualization were done in Perseus (v2.0.11)^52^, using biovenn^53^ for Venn diagrams or using custom scripts in R (v4.5.2) within R Studio (v2026.01.0.392) as well as Python (v 3.10.12) using the following libraries: Matplotlib: 3.7.1, Pandas: 2.0.3 and NumPy: 1.25.2. For volcano plot generation in Perseus, quantitative values were log2-transformed. For data on heat shock response on single B. subtilis *ΔmcsB* cells missing values were imputed using values from normal distribution (imputation into a left-shifted normal distribution; Perseus default parameters: width = 0.3, down-shift = 1.8, applied to the full matrix) and data were then Z-score normalized (per protein across samples). Differential testing used a two‑sided Student’s t-test with permutation-based multiple-testing control (250 randomizations) and a false discovery rate cutoff of 0.05. The S0 parameter^54^ was set to 0.1 to introduce an artificial within-group variance that balances fold-change and p‑value.

Troubleshooting and finetuning of custom scripts was supported by AI based LLM using Abacus (abacus.ai). All used code used was deposited on GitHub and is available via https://github.com/vbc-proteomics-org/bacSCP^55^. Workflow charts were generated using biorender. GO slim annotations were procured via the QuickGO browser (https://www.ebi.ac.uk/QuickGO/slimming)^56^, using the goslim_prokaryote_ribbon preset. Only hits assigned by Uniprot were taken, and redundant annotations removed.

### Reporting summary

Further information on research design is available in the Nature Portfolio Reporting Summary linked to this article.

## Supplementary information

Supplementary Information

Reporting Summary

Transparent Peer Review file

## Source data

Source Data

## Data Availability

The MS raw proteomics data, Spectronaut search results and FASTA files used have been deposited to the ProteomeXchange Consortium via the PRIDE^57^ partner repository with the dataset identifier PXD077117. Source data are provided with this paper.

## Code availability

Data analysis of Spectronaut search results and figure plotting are available on GitHub via link: https://github.com/vbc-proteomics-org/bacSCP^55^.
