# Supplementary material for: Pushing the limits of single-cell proteomics to investigate bacterial heterogeneity using bacSCP
Source: Nat Commun. 2026 Sep 1;17:10425. doi: 10.1038/s41467-026-77426-y (PMC13631272; doi:10.1038/s41467-026-77426-y)
Supplement: Supplementary file 1 [file 41467_2026_77426_MOESM1_ESM.pdf]

Pushing the limits of single-cell-proteomics to investigate bacterial heterogeneity using bacSCP.

Julia Leodolter[1], Tim Thierer[1], Karl Mechtler[1,2,3], Manuel Matzinger[1,§]

[1] Research Institute of Molecular Pathology (IMP), Vienna BioCenter (VBC), Vienna, Austria.
[2] Gregor Mendel Institute of Molecular Plant Biology (GMI), Austrian Academy of Sciences, Vienna BioCenter (VBC), Vienna, Austria.
[3] Institute of Molecular Biotechnology (IMBA), Austrian Academy of Sciences, Vienna BioCenter (VBC), Vienna, Austria.

§ Correspondence to: manuel.matzinger@imp.ac.at

Content

Supplementary Figure 1: **Correlation of protein quantity to quality metrics**

Supplementary Figure 2: **Overlap of quantified proteins from single E. coli vs 0 cell controls**

Supplementary Figure 3: **Representative extracted ion chromatograms for peptides found in negative controls as well as single cell samples**

Supplementary Figure 4: **Biological functions and cellular compartments of quantified proteins from E. coli**

Supplemental Figure 5: **Influence of precursors per protein and datapoints across peak on quantification accuracy**

Supplementary Figure 6: **Protein quantity distribution from single B. subtilis matched to bulk level analysis**

Supplementary Figure 7: **Investigation of heat shock response on single B. subtilis ΔmcsB *cells***

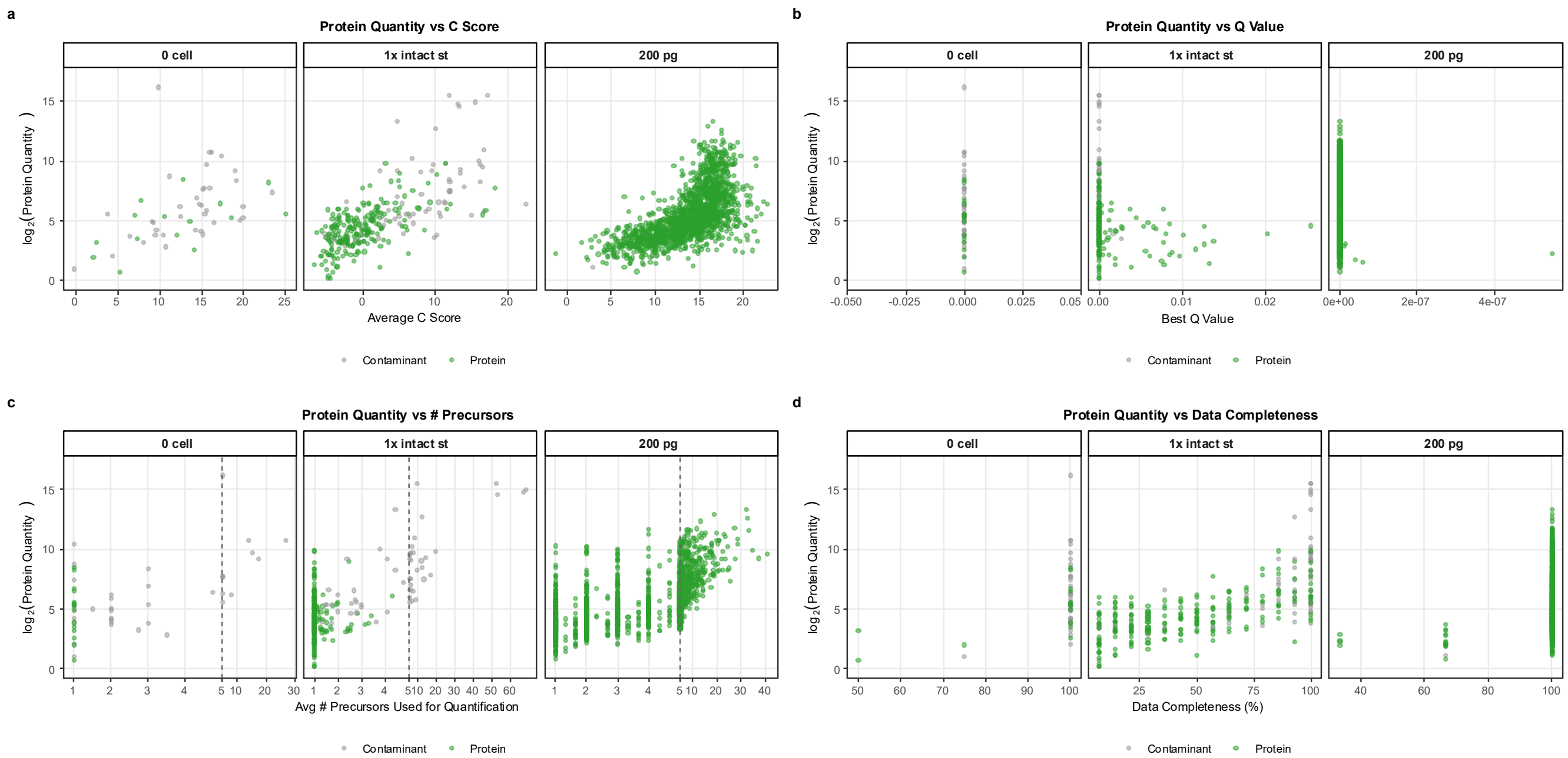

Supplementary Figure 1: ***Correlation of protein quantity to quality metrics****. Data of Figure 2, showing 0 cell controls (n=4), stained intact single E. coli (n = 14) and a 200 pg reference from diluted bulk E. coli peptides (n = 3). Dot plots show the correlation of average protein quantity to C Score (****a****), Q Value (****b****), the number of precursors used for quantification (****c****) or data completeness, meaning the fraction of replicates that protein was quantified in (****d****).*

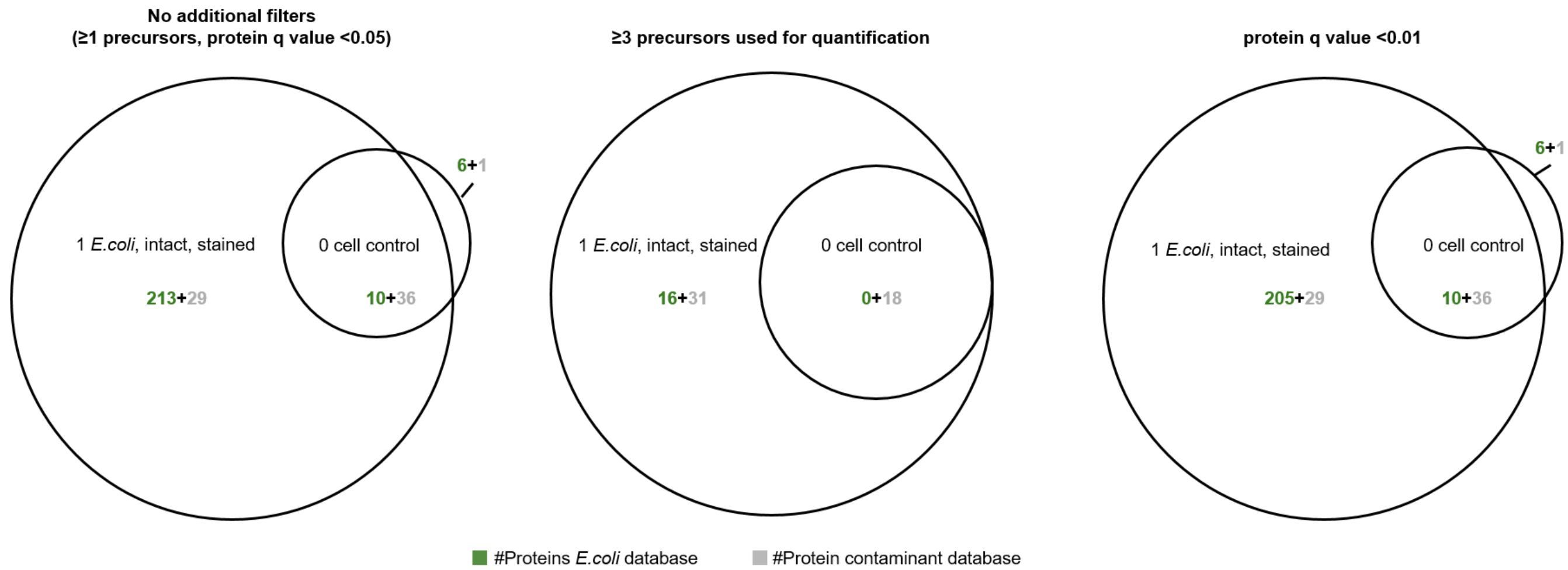

Supplementary Figure 2: ***Overlap of quantified proteins from single E. coli vs 0 cell controls****. Data of Figure 2 from intact stained single E. coli (n=14) and 0 cell controls (n=4). Venn diagrams show the overlap of quantified proteins after applying the indicated filtering strategy starting from default settings in Spectronaut (indicated as no additional filters) and with proteins from the CRAPome contaminant database shown in grey.*

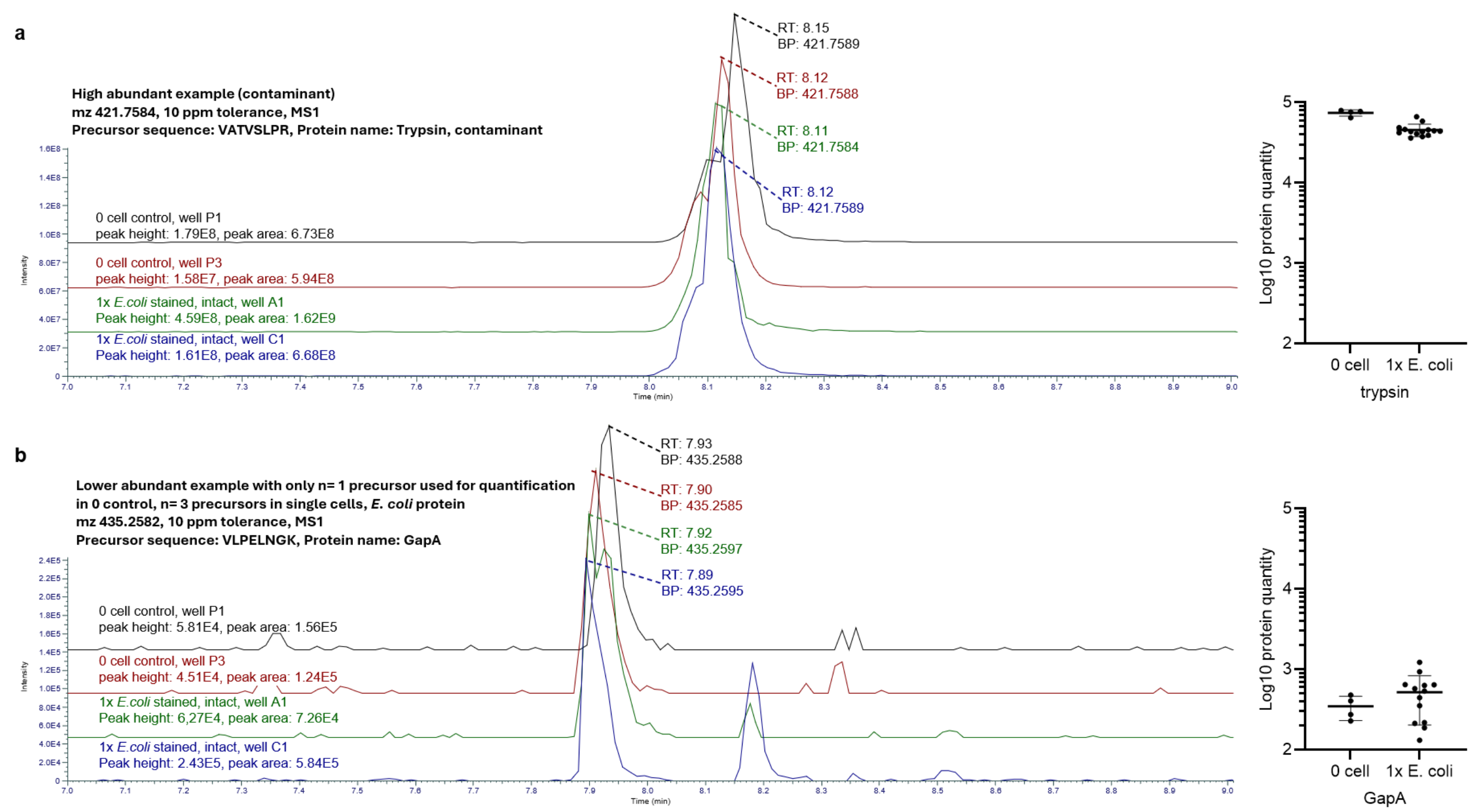

Supplementary Figure 3: ***Representative extracted ion chromatograms for peptides found in negative controls (from n=4 total negative control files) as well as single cell samples (from a total of n=14 single cell files)****. Data of Figure 2. Traces from 4 representative raw-files for a selected high abundant precursor (**a**) and low abundant precursor (**b**) as indicated. Plots on the right side show the quantitative distribution of the same precursor over all n=4 0 cell control samples and n=14 single cell samples respectively. Those plots show the median (center line) with error bars indicating the standard deviation and dots representing each individual value.*

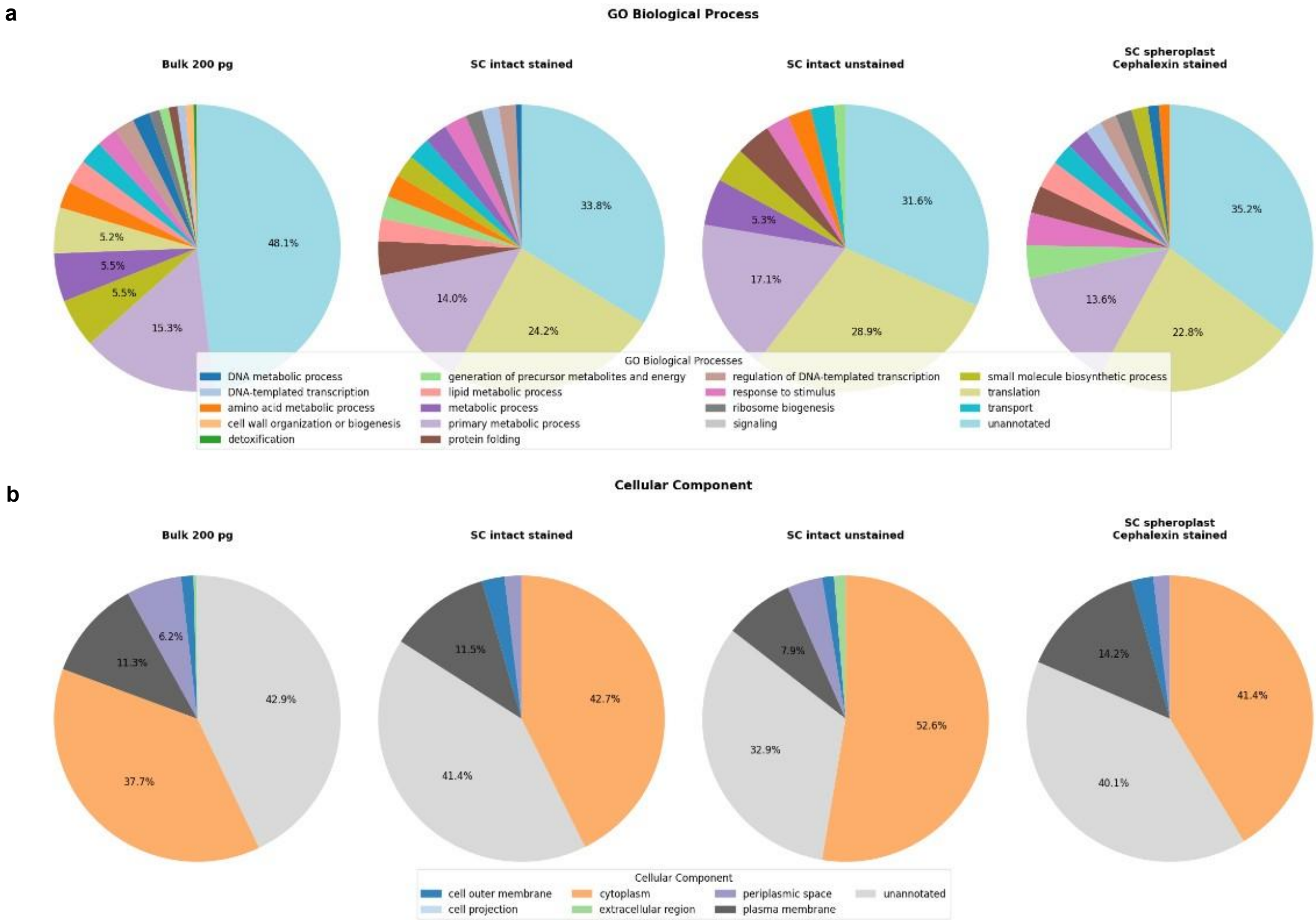

Supplementary Figure 4. ***Biological functions and cellular compartments of quantified proteins from E. coli.*** *GoSlim analysis of diluted bulk and single E. coli in dependence of sample preparation strategy as indicated. SC: single cell. Pie charts depict the biological processes (**a**) and cellular compartments (**b**) reported for those proteins.*

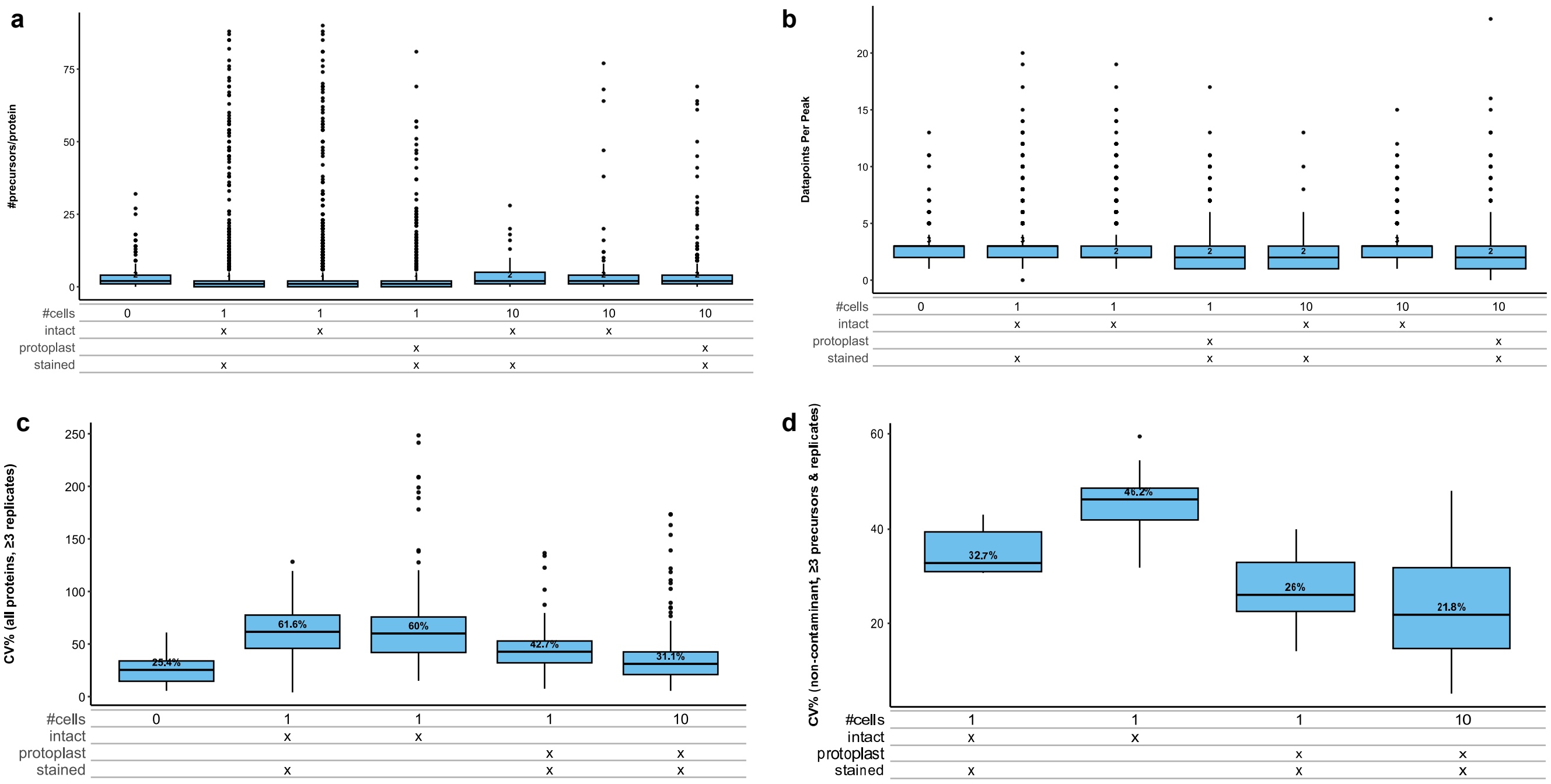

Supplementary Figure 5. ***Influence of precursors per protein and datapoints across peak on quantification accuracy*** *Data of Figure 3 is shown, the box-plots depict the distribution of number of precursors used for quantification per protein (**a**), the number of datapoints across the chromatographic elution peak used to quantify precursors on MS1 level (**b**), the CV on protein level including all proteins found in at least 3 replicates (**c**), or the CV of all bacterial proteins, excluding contaminants, found with at least 3 precursors and in 3 replicates (**d**). All boxplots show the median (center line), 25th and 75th percentile (box bounds), and whiskers extending to the largest/smallest value within 1.5x the interquartile range from the hinges*

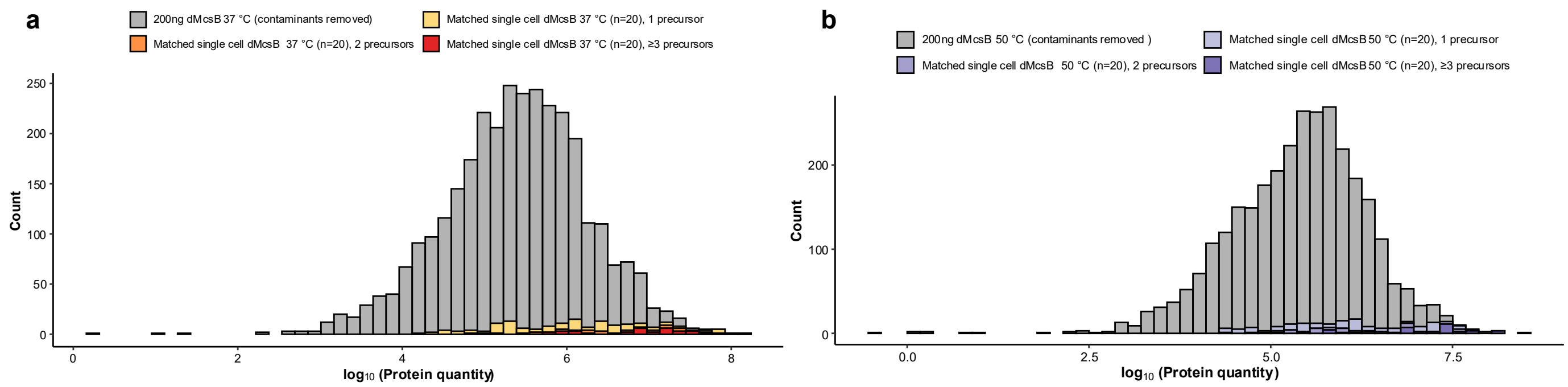

Supplementary Figure 6. ***Protein quantity distribution from single B. subtilis matched to bulk level analysis****. Histogram showing distribution of proteins found from 200 ng ΔmcsB B. subtilis and those from single cells matched to bulk quantities. Data shown for non-heat shocked, 37 °C (**a**) and heat shocked, 50 °C cells (**b**), respectively and are color coded depending on the number of precursors used for quantification for each protein.*

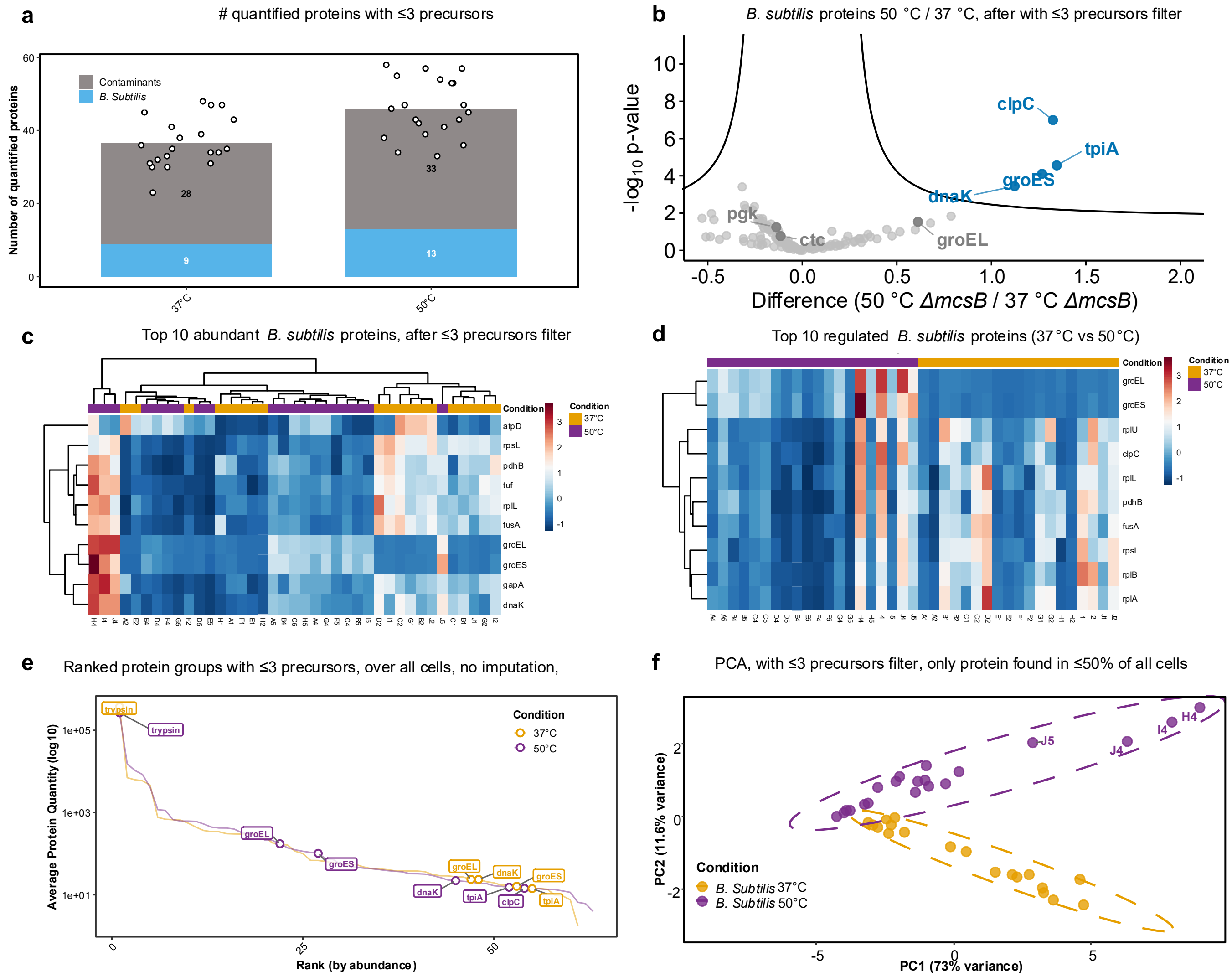

Supplementary Figure 7: **Investigation of heat shock response on single *B. subtilis ΔmcsB* cells.** *Data of Figure 6 but filtered for n ≥3 precursors used for quantification of each protein within each sample group.* ***a****. Bars indicate the average number of quantified protein groups from single B. subtilis with (50 °C) or without (37 °C) heat-shock, n=20 individual cells per condition.* ***b****: Volcano plot showing regulated non-contaminant proteins upon heat-shock.* ***c-d.*** *Heatmaps showing relative quantities of the top 10 abundant or top 10 upon heat-shock regulated proteins. P-values were calculated using a two-sided Student's t-test with permutation-based multiple-testing correction (FDR < 0.05); see Methods for details.* ***e.*** *By quantity ranked protein groups found in all n=20 single cells measured/condition with most regulated proteins as seen in B highlighted.* ***f.*** *Principal component analysis from heat-shocked (50 °C) and non-heat-shocked (37 °C) single B. subtilis measured.*
