# Supplementary material for: Pushing the limits of single-cell proteomics to investigate bacterial heterogeneity using bacSCP
Source: Nat Commun. 2026 Sep 1;17:10425. doi: 10.1038/s41467-026-77426-y (PMC13631272; doi:10.1038/s41467-026-77426-y)
Supplement: Supplementary file 2 [file 41467_2026_77426_MOESM2_ESM.pdf]

nature portfolio

Corresponding author(s): Manuel Matzinger

Last updated by author(s): Jul 3, 2026

Reporting Summary

Nature Portfolio wishes to improve the reproducibility of the work that we publish. This form provides structure for consistency and transparency in reporting. For further information on Nature Portfolio policies, see our Editorial Policies and the Editorial Policy Checklist.

Statistics

For all statistical analyses, confirm that the following items are present in the figure legend, table legend, main text, or Methods section.

n/a	Confirmed	
☐	☒	The exact sample size (*n*) for each experimental group/condition, given as a discrete number and unit of measurement
☐	☒	A statement on whether measurements were taken from distinct samples or whether the same sample was measured repeatedly
☐	☒	The statistical test(s) used AND whether they are one- or two-sided<br>*Only common tests should be described solely by name; describe more complex techniques in the Methods section.*
☐	☒	A description of all covariates tested
☐	☒	A description of any assumptions or corrections, such as tests of normality and adjustment for multiple comparisons
☐	☒	A full description of the statistical parameters including central tendency (e.g. means) or other basic estimates (e.g. regression coefficient) AND variation (e.g. standard deviation) or associated estimates of uncertainty (e.g. confidence intervals)
☐	☒	For null hypothesis testing, the test statistic (e.g. *F*, *t*, *r*) with confidence intervals, effect sizes, degrees of freedom and *P* value noted<br>*Give P values as exact values whenever suitable.*
☒	☐	For Bayesian analysis, information on the choice of priors and Markov chain Monte Carlo settings
☐	☒	For hierarchical and complex designs, identification of the appropriate level for tests and full reporting of outcomes
☒	☐	Estimates of effect sizes (e.g. Cohen's *d*, Pearson's *r*), indicating how they were calculated

Our web collection on statistics for biologists contains articles on many of the points above.

Software and code

Policy information about availability of computer code

Data collection	Mass spectrometry data was acquired using the Orbitrap Astral MS, using Thermo Tune software (version: 2.1). Cell isolation including the recording of sizes and fluorescence signals was done using the cellenONE using its control software (v2.0-1143).
Data analysis	Proteomics data was analysed using Spectronaut (v. 20.4.260109.92449). Statistical analysis and visualization were done in Perseus (v2.0.11), using biovenn for Venn diagrams or using custom scripts in R (v4.5.2) within R Studio (v2026.01.0.392) as well as Python (v 3.10.12) using the following libraries: Matplotlib: 3.7.1, Pandas: 2.0.3 and NumPy: 1.25.2. For volcano plot generation in Perseus, quantitative values were log2-transformed and missing values were imputed using values from normal distribution" (imputation into a left-shifted normal distribution; Perseus default parameters: width = 0.3, down-shift = 1.8, applied to the full matrix). Data were then Z-score normalized (per protein across samples). Differential testing used a two-sided Student's t-test with permutation-based multiple-testing control (250 randomizations) and a false discovery rate cutoff of 0.05. The S0 parameter54 was set to 0.1 to introduce an artificial within-group variance that balances fold-change and p-value.<br>Troubleshooting and finetuning of custom scripts was supported by AI based LLM using Abacus (abacus.ai). All used code used was deposited on GitHub and is available via https://github.com/vbc-proteomics-org/bacSCP.

For manuscripts utilizing custom algorithms or software that are central to the research but not yet described in published literature, software must be made available to editors and reviewers. We strongly encourage code deposition in a community repository (e.g. GitHub). See the Nature Portfolio guidelines for submitting code & software for further information.

Data

Policy information about availability of data

All manuscripts must include a data availability statement. This statement should provide the following information, where applicable:

- Accession codes, unique identifiers, or web links for publicly available datasets
- A description of any restrictions on data availability
- For clinical datasets or third party data, please ensure that the statement adheres to our policy

The MS raw proteomics data, Spectronaut search results and FASTA files used have been deposited to the ProteomeXchange Consortium via the PRIDE partner repository with the dataset identifier PXD077117.

Research involving human participants, their data, or biological material

Policy information about studies with human participants or human data. See also policy information about sex, gender (identity/presentation), and sexual orientation and race, ethnicity and racism.

Reporting on sex and gender	no human participants
Reporting on race, ethnicity, or other socially relevant groupings	no human participants
Population characteristics	no human participants
Recruitment	no human participants
Ethics oversight	no human participants

Note that full information on the approval of the study protocol must also be provided in the manuscript.

Field-specific reporting

Please select the one below that is the best fit for your research. If you are not sure, read the appropriate sections before making your selection.

[x] Life sciences   [ ] Behavioural & social sciences   [ ] Ecological, evolutionary & environmental sciences

For a reference copy of the document with all sections, see nature.com/documents/nr-reporting-summary-flat.pdf

Life sciences study design

All studies must disclose on these points even when the disclosure is negative.

Sample size	No sample size calculation was performed. As we saw a very low variance between n=3 measured technical replicates technical variability of our LC-MS methodology was proven low even for inputs at single bacteria level and we decided that no further replicates were needed.<br>For single-cell experiments 14 E.coli and 20 Bacillus Subtilis cells per condition were compared. 4 blank controls were included in every single-cell study.<br>No sample size calculation was performed, but heterogeneity between cell types/cells was clearly visible with the number of measured replicates, indicating it is sufficient. We assume, more cell replicates would benefit the study, but since this is a proof of concept study were limited in given measurement time.
Data exclusions	No replicate measurement was excluded. quantified proteins from the resulting Data were excluded if they did not pass a FDR treshold of 1% . for some graphs additional quality filters such as a minimum number of precursors used for quantification (1-3) or a minimum data completeness across the measured single cells of 50% were applied. Additional filters are annotated accordingly in each figure legend.
Replication	No replicate measurements from single cells are possible as the entire sample is used by a single measurement. Hence individual cells are considered replicates and n=14-20 cells were measured per condition, as given in each figure legend. For negative controls (0 cell samples) n= 4 replicates were measured. All bulk samples used 3 replicates. All replicate measurements were considered successful, with no replicated being excluded.
Randomization	Single Cell samples were isolated within a given range of cell-size and fluorescence intensity without any other means of sorting, which initiated randomization by experimental design . Technical replicates were used for positive controls (bulk samples), and randomization was considered not relevant for those as we focused on improving and validating the capabilities of our workflow rather than comparing any sample treatments. Also no clinical samples were used in this study.
Blinding	No blinding was performed or relevant to this work. Knowledge sample-types compared in this study was required to check if our developed workflow delivers results as expected as well as for quality control checks and method optimization.

Reporting for specific materials, systems and methods

We require information from authors about some types of materials, experimental systems and methods used in many studies. Here, indicate whether each material, system or method listed is relevant to your study. If you are not sure if a list item applies to your research, read the appropriate section before selecting a response.

Materials & experimental systems

n/a	Involved in the study
☒	☐ Antibodies
☒	☐ Eukaryotic cell lines
☒	☐ Palaeontology and archaeology
☒	☐ Animals and other organisms
☒	☐ Clinical data
☒	☐ Dual use research of concern
☒	☐ Plants

Methods

n/a	Involved in the study
☒	☐ ChIP-seq
☒	☐ Flow cytometry
☒	☐ MRI-based neuroimaging

Plants

Seed stocks	n.a., bacteria were used
Novel plant genotypes	n.a., bacteria were used
Authentication	n.a., bacteria were used
