# Supplementary material for: Pushing the limits of single-cell proteomics to investigate bacterial heterogeneity using bacSCP
Source: Nat Commun. 2026 Sep 1;17:10425. doi: 10.1038/s41467-026-77426-y (PMC13631272; doi:10.1038/s41467-026-77426-y)
Supplement: Supplementary file 3 [file 41467_2026_77426_MOESM3_ESM.pdf]

nature portfolio

Peer Review File

Pushing the limits of single-cell-proteomics to investigate bacterial heterogeneity using bacSCP.

Corresponding Author: Dr Manuel Matzinger

This file contains all reviewer reports in order by version, followed by all author rebuttals in order by version.

Version 0:

Reviewer comments:

Reviewer #1

(Remarks to the Author)
Leodolter et al. present bacSCP, a proof-of-concept extension of single-cell proteomics (SCP) to single bacteria. This is an inherently challenging target given that bacteria contain roughly 1000-fold less protein than mammalian cells. Combining image- and fluorescence-gated cellenONE isolation, a One-Pot freeze-thaw lysis/digestion workflow, short LC gradients on an IonOpticks column and label-free DIA on an Orbitrap Astral, the authors quantify ~50–65 bacterial proteins per single E. coli or B. subtilis cell. Most importantly, they detect biologically meaningful upregulation of the GroEL/GroES and ClpC chaperones upon heat shock at single-cell resolution in a B. subtilis ΔmcsB background, with suggestive evidence of population heterogeneity in the stress response. To my knowledge this is the first label-free SCP demonstration on single bacteria and a clear step forward from the prior TMT carrier-channel attempt (Végvári et al., 2023). Overall, this is a well-executed and honest proof-of-concept that establishes label-free SCP of single bacteria for the first time. The technical advance is clear, the biological readout is meaningful, the authors are appropriately cautious about the limits of the inferences they draw, and data and code are properly deposited. I recommend acceptance after minor revisions.

Noteworthy results and significance. The technical achievement, i.e. robust label-free quantification of tens of bacterial proteins from single cells without a carrier proteome, is the central contribution and a genuine advance over the only previously published attempt at bacterial SCP. The biological readout (induction of GroEL/ES and ClpC, accompanied by Ctc and Pgk) is well chosen, both because the regulation is large and because it is interpretable in the context of the well-studied B. subtilis heat-shock response and the McsB/YwlE pArg axis. The work will be of interest to both the SCP and bacterial physiology communities and lays a useful foundation for future studies of antibiotic persistence and bacterial bet-hedging at proteome resolution. I would also like to commend the authors for using Spectronaut v20 rather than v19; the latter has been observed to produce inflated identification numbers at low input, and the choice of v20 is a responsible methodological decision in the context of an ultra-low-input proof-of-concept.

Literature coverage. The eukaryotic SCP context is reasonably covered, but a few key references in the label-free DIA-SCP space could strengthen the comparison. I would recommend citing Zhu et al. (Nat Commun 2018) as the foundational nanoPOTS paper for sub-microlitre trace-sample processing, and Cong et al. (Chem Sci 2021) for the ultrasensitive Orbitrap + FAIMS label-free workflow at >1,000 protein groups per single mammalian cell. both of which are directly relevant precedents for the technical choices made here. Petrosius et al. (Mol Cell Proteomics 2025) should also be considered, as this work characterises the boundaries of accurate quantification on the Orbitrap Astral in an SCP context.

Support for the conclusions. The chaperone induction claim is solidly supported by both bulk titration (Fig. 4) and single-cell data (Fig. 6B,E). The "heterogeneity" claim is a little speculative since the strong-responder subpopulation comprises ~4/20 cells, but the authors are appropriately cautious and explicitly note that biological versus technical variance cannot yet be disentangled at this sample size. For a proof-of-concept I find this framing fair and no additional experiments seem essential. The framing "we demonstrate that bacSCP is applicable across both Gram-positive and Gram-negative bacteria" (Discussion) is technically supported for the two model organisms tested (E. coli and B. subtilis), but it slightly extrapolates beyond the experimental scope. I would suggest softening to e.g. "applicable to both E. coli and B. subtilis, representing Gram-negative and Gram-positive model organisms respectively", since species with more refractory walls (mycobacteria, mycoplasma, encapsulated pathogens) are likely to be even more challenging.

Methodology and reproducibility. The methods are sound and well-suited to the state of the art. The systematic comparison

of intact vs. protoplast/spheroplast preparations, with and without Hoechst staining, is informative, and the fluorescence-based gating to reach near-100% confidence on bacterial isolation is a useful practical contribution. Reporting on cellenONE parameters, gradient, FAIMS settings, and Spectronaut version is sufficient for reproduction, and reproducibility is well supported by the deposition of raw files, Spectronaut search outputs and FASTA files on PRIDE (PXD077117) and of the analysis code on GitHub (vbc-proteomics-org/bacSCP).

Minor points and typos.
• The cross-reference in the Results description of Figure 6F ("Error! Reference source not found.F") should be resolved.
• "contaminats" (Results, ~line 133) → "contaminants".
• "B. subitilis" (Results section "Heat shock response", two occurrences) → "B. subtilis".
• "Generation of protoplast/spheroblast cells" (Methods header) → "spheroplast".
• Table 1 caption: "Schmid et al.30" should read "Schmidt et al." to match reference 30.
• “Orbitrap Fusion Eclipse MS” should be corrected to ”Orbitrap Eclipse MS”
• “Principial Component Analysis” should be corrected to ”Principal Component Analysis”
• Minor: in Fig. 3C the lower CV in the 0-cell control versus single cells is worth a single sentence of explicit interpretation (noise-dominated, narrow Gaussian distribution rather than peptide signal). The reduced physiological relevance of cephalexin-enlarged spheroplasts is well acknowledged; the data nonetheless usefully define the lower size limit accessible on the cellenONE.

(Remarks on code availability)

Reviewer #2

(Remarks to the Author)
Summary
This manuscript presents bacSCP, a label-free single-bacterium proteomics workflow aimed at extending recent advances in single-cell proteomics to bacterial cells. The work addresses an important analytical challenge: individual bacterial cells contain far less protein than mammalian cells, are difficult to lyse efficiently, and are highly susceptible to contamination and sample-loss effects. The authors optimize sorting, lysis, and LC–MS/MS acquisition strategies for E. coli and B. subtilis, ultimately reporting quantification of tens of bacterial proteins per single bacterium and using the workflow to detect heat-shock-associated protein changes in B. subtilis ΔmcsB cells. The central biological result is the detection of increased GroEL, GroES, and ClpC abundance after heat shock, with the authors further suggesting possible cell-to-cell heterogeneity in the heat-shock response. These findings are technically interesting and potentially important, because the study provides a proof-of-concept route toward bacterial single-cell proteomics without a carrier proteome, library, or isobaric multiplexing. The manuscript is generally well motivated and addresses a timely area in proteomics, but several aspects of the identification confidence, contaminant handling, and interpretation of low-abundance single-cell measurements require further clarification before the conclusions are fully supported. I suggest a revision of the manuscript before acceptance.

Major points
1. Confidence of low-abundance bacterial identifications in single cells
In Figure 2B and Supplemental Figure 6, the proteins detected at the single-bacterium level appear to extend into relatively low-abundance regions of the matched bulk distributions. In single-cell or very-low-input proteomics, I would generally expect the proteins confidently detected in single cells to be strongly enriched among the highest-abundance proteins, often forming a high-abundance-biased distribution relative to bulk. The broad lower-intensity tail raises the possibility that some proteins below approximately 2.75 log10 protein quantity/intensity may represent low-confidence identifications, carryover, noise-driven quantification, or false assignments.

The authors should provide additional evidence supporting the confidence of these low-abundance bacterial protein identifications. Helpful analyses would include, for proteins above and below this approximate abundance threshold: number of unique peptides, number of precursors, precursor q-values, protein q-values, fragment-ion evidence, chromatographic peak quality, retention-time consistency, missingness across cells, presence in zero-cell controls, and whether the identifications depend on any transfer/matching between runs. The authors already show that filtering for proteins quantified with at least three precursors removes bacterial hits from zero-cell controls and improves median CV in single-cell samples; applying this or a similar filter directly to Figure 2B and Supplemental Figure 6 would make the identification-confidence argument much stronger.

2. Contaminant and zero-cell protein identifications need deeper characterization
The manuscript reports that up to 98% of total detected protein quantity in single-cell samples originates from contaminants, largely because of the very low bacterial protein input and the large excess of trypsin required for sample preparation. This is an important and unavoidable challenge, but it also means that contamination and misassignment must be rigorously addressed.

Given the low CVs observed for apparent bacterial proteins detected in samples that did not contain bacteria, were those bacterial proteins identified in zero-cell controls excluded from all downstream analyses? The authors hypothesize that zero-cell bacterial signals are dominated by noise rather than true peptides, but this should be substantiated with additional metrics.

The authors searched against bacterial proteomes plus the CRAPome contaminant database. However, given the

prominent contribution of human-derived contaminants such as keratins, it would be useful to know whether the data were ever searched against a full human proteome database, or a combined bacterial-human-contaminant database, to estimate the extent of human contamination and potential peptide misassignment.

The authors should also clarify whether any bacterial peptides overlap with human peptides, whether such peptides were removed, and how consistent contaminant protein measurements were compared with bacterial protein measurements.

A direct comparison of CV distributions for bacterial proteins versus contaminant proteins would help determine whether the bacterial measurements behave like true low-input biological signals or more like technical/background signals.

3. Clarify FDR control and DirectDIA identification strategy at the single-cell level
The data were searched library-free using Spectronaut DirectDIA+, with replicates defined as the same condition, and against species-specific bacterial databases plus CRAPome. Because the manuscript's conclusions rely on very low numbers of detected proteins per cell, it is important to understand how identification transfer, replicate grouping, and FDR control affect the reported single-cell identifications.

The authors should clarify whether identifications were controlled at the run, precursor, peptide, and protein levels independently for each single-cell run, or whether evidence was shared across replicates/conditions. Were zero-cell controls included in the same search?

Minor points
1. LC–MS method duration / 80 samples per day
The authors use an 80 SPD method on the Orbitrap Astral, with a short active gradient. Since sensitivity is the central limitation in this study, it would be useful to know whether the authors tested slower methods, such as 40–60 SPD, and whether longer gradients or different DIA window schemes improved low-abundance bacterial protein detection. Even a brief statement that this was not tested, or that 80 SPD was chosen to balance depth and throughput, would be helpful.

2. Definition of "quantified proteins"
The manuscript alternates between "proteins," "protein groups," "non-contaminant proteins," and "quantified proteins." The authors should define these consistently. For example, are all reported counts protein groups? Are one-peptide protein groups included? Are contaminants excluded from the headline numbers throughout? This is especially important for Figures 2, 3, and 6.

Textual and grammatical issues
1. The supplemental figure numbering appears incorrect. The supplement contents list Supplemental Figures 1–3, but the legends label the figures as Supplemental Figures 4–6.
2. Around line 132, the text refers to "Figure 1C" when discussing summed protein quantity and diluted bulk comparisons. This appears to refer to Figure 2C, not Figure 1C.
3. The text contains "Error! Reference source not found.F" when discussing the PCA of heat-shocked and non-heat-shocked cells. This should likely be corrected to Figure 6F.
4. "384 well pate" should be "384-well plate."
5. "contaminats" should be "contaminants."
6. "Principial Component Analysis" should be "Principal Component Analysis."
7. Please check spelling and formatting of strain names throughout, including instances such as "B. subitilis" and possible inconsistencies in ΔywlE/ΔywIE notation.

(Remarks on code availability)

Reviewer #3

(Remarks to the Author)
I am generally enthusiastic about this work. Not only is it a monumental achievement to convincingly demonstrate protein measurement from individual bacteria, but the authors made inroads toward showing the utility of the workflow for biological inquiry. Minor comments:
1. The authors should add citations to the first sentence of the introduction
2. Line 35, the CellenONE is not the only means of isolating single cells. The authors should also mention and cite works using FACS and Tecan UNO, also perhaps LCM.
3. Line 85, mention not only active gradients, but samples per day or cycle time.
4. Figure 2B. Clarify the x axis units. I don't suppose this is copy number? If it were, I don't think it would make sense. Please attempt to convert the protein quantity to detection limit in terms of copy number. This will be helpful for understanding whether the proteins measured below ~2 on the x axis make sense.

(Remarks on code availability)
NA

Version 1:

Reviewer comments:

Reviewer #2

(Remarks to the Author)
I thank the authors for their detailed responses. All of my concerns and questions have been answered and I have no further comments.

(Remarks on code availability)
I checked on the code which details how the figures were created, it all seems to be in line with expectations.

Open Access This Peer Review File is licensed under a Creative Commons Attribution 4.0 International License, which permits use, sharing, adaptation, distribution and reproduction in any medium or format, as long as you give appropriate credit to the original author(s) and the source, provide a link to the Creative Commons license, and indicate if changes were made.
In cases where reviewers are anonymous, credit should be given to 'Anonymous Referee' and the source.
The images or other third party material in this Peer Review File are included in the article's Creative Commons license, unless indicated otherwise in a credit line to the material. If material is not included in the article's Creative Commons license and your intended use is not permitted by statutory regulation or exceeds the permitted use, you will need to obtain permission directly from the copyright holder.
To view a copy of this license, visit https://creativecommons.org/licenses/by/4.0/

Reviewer #1 (Remarks to the Author):

Leodolter et al. present bacSCP, a proof-of-concept extension of single-cell proteomics (SCP) to single bacteria. This is an inherently challenging target given that bacteria contain roughly 1000-fold less protein than mammalian cells. Combining image- and fluorescence-gated cellenONE isolation, a One-Pot freeze-thaw lysis/digestion workflow, short LC gradients on an IonOpticks column and label-free DIA on an Orbitrap Astral, the authors quantify ~50–65 bacterial proteins per single E. coli or B. subtilis cell. Most importantly, they detect biologically meaningful upregulation of the GroEL/GroES and ClpC chaperones upon heat shock at single-cell resolution in a B. subtilis ΔmcsB background, with suggestive evidence of population heterogeneity in the stress response. To my knowledge this is the first label-free SCP demonstration on single bacteria and a clear step forward from the prior TMT carrier-channel attempt (Végvári et al., 2023). Overall, this is a well-executed and honest proof-of-concept that establishes label-free SCP of single bacteria for the first time. The technical advance is clear, the biological readout is meaningful, the authors are appropriately cautious about the limits of the inferences they draw, and data and code are properly deposited. I recommend acceptance after minor revisions.

We thank the reviewer for thoughtfully going through our manuscript and their positive feedback acknowledging the potential value of our study to the community and constructive comments for further improvements. Please find our detailed responses below.

Noteworthy results and significance. The technical achievement, i.e. robust label-free quantification of tens of bacterial proteins from single cells without a carrier proteome, is the central contribution and a genuine advance over the only previously published attempt at bacterial SCP. The biological readout (induction of GroEL/ES and ClpC, accompanied by Ctc and Pgk) is well chosen, both because the regulation is large and because it is interpretable in the context of the well-studied B. subtilis heat-shock response and the McsB/YwlE pArg axis. The work will be of interest to both the SCP and bacterial physiology communities and lays a useful foundation for future studies of antibiotic persistence and bacterial bet-hedging at proteome resolution. I would also like to commend the authors for using Spectronaut v20 rather than v19; the latter has been observed to produce inflated identification numbers at low input, and the choice of v20 is a responsible methodological decision in the context of an ultra-low-input proof-of-concept.

Literature coverage. The eukaryotic SCP context is reasonably covered, but a few key references in the label-free DIA-SCP space could strengthen the comparison. I would

recommend citing Zhu et al. (Nat Commun 2018) as the foundational nanoPOTS paper for sub-microlitre trace-sample processing, and Cong et al. (Chem Sci 2021) for the ultrasensitive Orbitrap + FAIMS label-free workflow at >1,000 protein groups per single mammalian cell. both of which are directly relevant precedents for the technical choices made here. Petrosius et al. (Mol Cell Proteomics 2025) should also be considered, as this work characterises the boundaries of accurate quantification on the Orbitrap Astral in an SCP context.

In line with the reviewers' recommendation, we added the nanoPOTS paper and FAIMS paper to highlight their contribution in improving sensitivity for low input samples as well as the work from Petrosius et al showcasing quantitative performance of the Astral MS.

Action Taken: Additional literature was included in our introduction as suggested acknowledging their contribution to trace-sample processing & analysis.

Support for the conclusions. The chaperone induction claim is solidly supported by both bulk titration (Fig. 4) and single-cell data (Fig. 6B,E). The "heterogeneity" claim is a little speculative since the strong-responder subpopulation comprises ~4/20 cells, but the authors are appropriately cautious and explicitly note that biological versus technical variance cannot yet be disentangled at this sample size. For a proof-of-concept I find this framing fair and no additional experiments seem essential. The framing "we demonstrate that bacSCP is applicable across both Gram-positive and Gram-negative bacteria" (Discussion) is technically supported for the two model organisms tested (E. coli and B. subtilis), but it slightly extrapolates beyond the experimental scope. I would suggest softening to e.g. "applicable to both E. coli and B. subtilis, representing Gram-negative and Gram-positive model organisms respectively", since species with more refractory walls (mycobacteria, mycoplasma, encapsulated pathogens) are likely to be even more challenging.

We agree with the reviewer that other species such as mycobacteria and others might be even more challenging.

Action Taken: We adopted phrasing as suggested.

Methodology and reproducibility. The methods are sound and well-suited to the state of the art. The systematic comparison of intact vs. protoplast/spheroplast preparations, with and without Hoechst staining, is informative, and the fluorescence-based gating to reach near-100% confidence on bacterial isolation is a useful practical contribution. Reporting on cellenONE parameters, gradient, FAIMS settings, and Spectronaut version is sufficient for reproduction, and reproducibility is well supported by the deposition of raw files, Spectronaut search outputs and FASTA files on PRIDE (PXD077117) and of the analysis code on GitHub (vbc-proteomics-org/bacSCP).

We thank the reviewer for their positive feedback on methodology!

Minor points and typos.

We are grateful to the referee for their attention to detail. All minor issues regarding formatting, typos, and references have been addressed in the revised manuscript.

• The cross-reference in the Results description of Figure 6F ("Error! Reference source not found.F") should be resolved.

We corrected this broken figure-link and checked for remaining broken links.

• "contaminats" (Results, ~line 133) → "contaminants".

We corrected this.

• "B. subitilis" (Results section "Heat shock response", two occurrences) → "B. subtilis".

We corrected this.

• "Generation of protoplast/spheroblast cells" (Methods header) → "spheroplast".

We corrected this.

• Table 1 caption: "Schmid et al.30" should read "Schmidt et al." to match reference 30.

We corrected this.

• "Orbitrap Fusion Eclipse MS" should be corrected to "Orbitrap Eclipse MS"

We corrected this.

• "Principial Component Analysis" should be corrected to "Principal Component Analysis"

We corrected this.

• Minor: in Fig. 3C the lower CV in the 0-cell control versus single cells is worth a single sentence of explicit interpretation (noise-dominated, narrow Gaussian distribution rather than peptide signal). The reduced physiological relevance of cephalexin-enlarged spheroplasts is well acknowledged; the data nonetheless usefully define the lower size limit accessible on the cellenONE.

We thank the reviewer for their suggestion and fully agree that 0 cell controls are noise-dominated.

Action Taken: We checked in the manuscript results section, and for Figure 3C adopted the existing description as follows and in line with the reviewer's suggestion:

“We noted that the CV for the zero-cell negative control seems lower compared to single cells, although no bacterial input is present. We hypothesize that the signal in the negative control is dominated by noise rather than signals from true peptides. This observation might be reasoned by the narrower Gaussian distribution of noise levels due to the lack of biological variation.”

Reviewer #2 (Remarks to the Author):

Summary
This manuscript presents bacSCP, a label-free single-bacterium proteomics workflow aimed at extending recent advances in single-cell proteomics to bacterial cells. The work addresses an important analytical challenge: individual bacterial cells contain far less protein than mammalian cells, are difficult to lyse efficiently, and are highly susceptible to contamination and sample-loss effects. The authors optimize sorting, lysis, and LC–MS/MS acquisition strategies for E. coli and B. subtilis, ultimately reporting quantification of tens of bacterial proteins per single bacterium and using the workflow to detect heat-shock-associated protein changes in B. subtilis ΔmcsB cells. The central biological result is the detection of increased GroEL, GroES, and ClpC abundance after heat shock, with the authors further suggesting possible cell-to-cell heterogeneity in the heat-shock response. These findings are technically interesting and potentially important, because the study provides a proof-of-concept route toward bacterial single-cell proteomics without a carrier proteome, library, or isobaric multiplexing. The manuscript is generally well motivated and addresses a timely area in proteomics, but several aspects of the identification confidence, contaminant handling, and interpretation of low-abundance single-cell measurements require further clarification before the conclusions are fully supported. I suggest a revision of the manuscript before acceptance.

We thank the reviewer for their positive feedback confirming the technical advancements made and potential importance of the presented work. We acknowledge the importance of the questions raised to fully support our conclusions and to further improve the quality of this study. Please find our detailed responses below.

Major points

1. Confidence of low-abundance bacterial identifications in single cells
In Figure 2B and Supplemental Figure 6, the proteins detected at the single-bacterium level appear to extend into relatively low-abundance regions of the matched bulk distributions. In single-cell or very-low-input proteomics, I would

generally expect the proteins confidently detected in single cells to be strongly enriched among the highest-abundance proteins, often forming a high-abundance-biased distribution relative to bulk. The broad lower-intensity tail raises the possibility that some proteins below approximately 2.75 log10 protein quantity/intensity may represent low-confidence identifications, carryover, noise-driven quantification, or false assignments.

We thank the reviewer for raising this important question and confirm their assumption that low abundant proteins found are predominantly sparse ones. Their confidence seems high by means of very low q values (as given by Spectronaut) and no matching to higher input libraries or imputation has been performed either. However, only those proteins quantified with less than 3 precursors are amongst the low abundant ones. When filtering for a minimum of 2- 5 precursors used for quantification of specific proteins, the shift towards the high abundant fraction of the minibulk reference becomes more obvious the more stringent we filter. Of note, the minibulk referenced is from 200pg, an input expected in an average sized mammalian cell. Since we compare here low input to very low input, we decided to additionally check how the abundance distribution looks compared to higher inputs of 0.2, 2, 20 or 200 ng from a commercial *E. coli* digest respectively. In line with our expectation, we found that higher inputs yielded coverage of lower abundant proteins. However, this shift is less pronounced for lower inputs compared against each other (i.e. from 2ng to 200pg), supporting that a shift towards higher abundant proteins is expected for single cells but not to the same extend as seen in the higher input range which seems to unlock detectability of more protein slow abundant within the sample mixture. This data is shown in Point to Point Figure 1.

Action Taken: We adopted Figure 2B and Supplementary Figure 6, now showing proteins with 1, 2 or 3 and more precursors used for quantification.

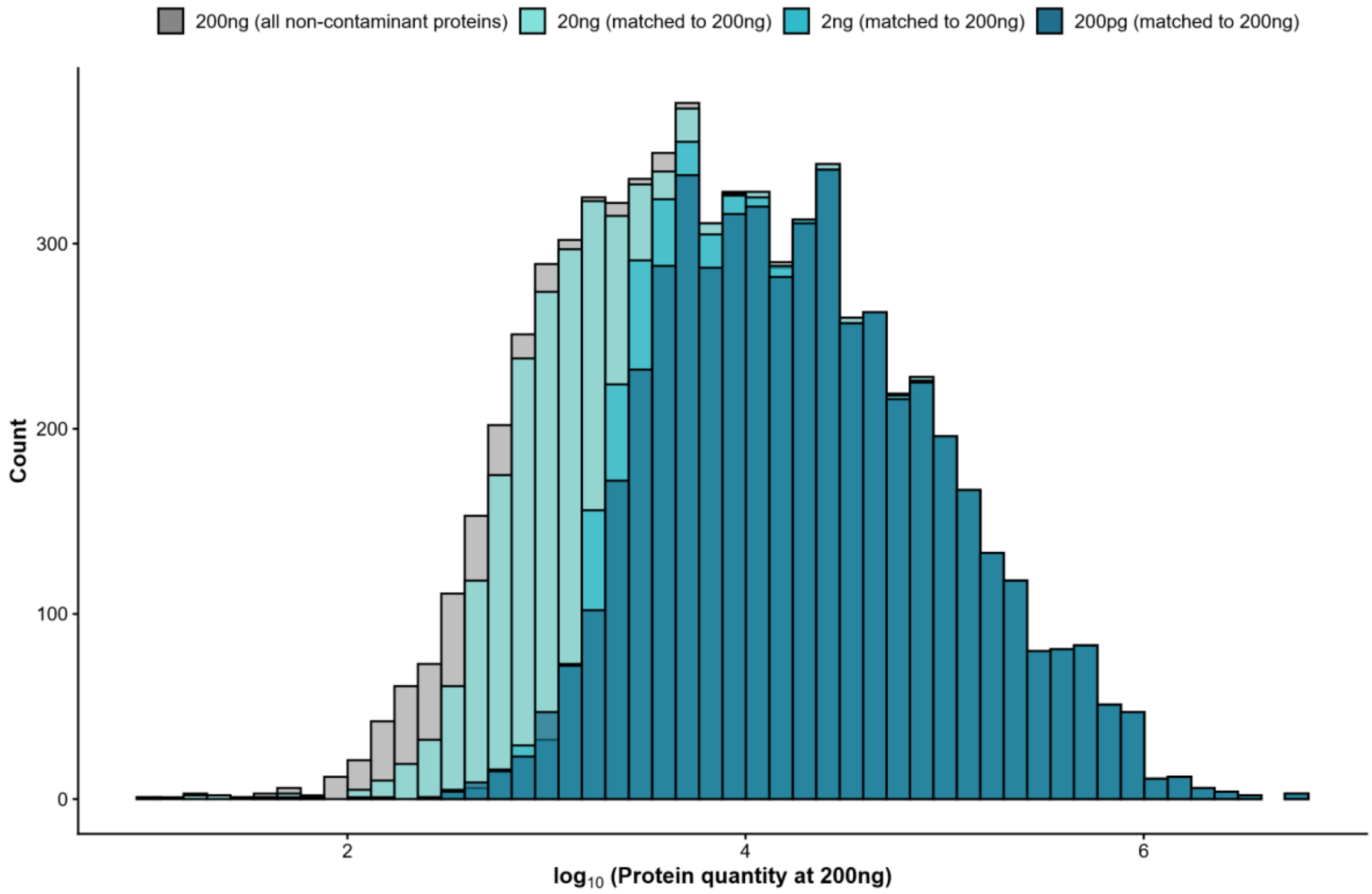

Point_to_Point Figure 1: Histogram showing the distribution of E. coli protein quantities measured at 200 ng bulk input (gray). Colored overlays represent the 200 ng quantities of proteins that were also detected at lower bulk inputs (20 ng, 2 ng, 200 pg) illustrating which fraction of the 200 ng dynamic range is accessible at each input amount

2. The authors should provide additional evidence supporting the confidence of these low-abundance bacterial protein identifications. Helpful analyses would include, for proteins above and below this approximate abundance threshold: number of unique peptides, number of precursors, precursor q-values, protein q-values, fragment-ion evidence, chromatographic peak quality, retention-time consistency, missingness across cells, presence in zero-cell controls, and whether the identifications depend on any transfer/matching between runs. The authors already show that filtering for proteins quantified with at least three precursors removes bacterial hits from zero-cell controls and improves median CV in single-cell samples; applying this or a similar filter directly to Figure 2B and Supplemental Figure 6 would make the identification-confidence argument much stronger.

In line with the findings described in our response to the previous question, and the reviewer's assumption, those low abundant proteins were quantified based on only a single precursor. It is correct, in the 0 cell control all bacterial proteins are very low abundant hence are filtered away when filtering for a minimum of 3 precursors. Our data was analyzed in method evaluation mode with separate conditions defined for each sample type, hence no MBR/transfer was active towards any higher input samples.

Of note Spectronaut reports proteins with q value <0.05 (so confidently identified) but not necessarily quantified. Applying a more stringent q value

filter at q<0.01 on protein level introduces only minor changes to the result (See new Supplemental Figure 2) and we observed no correlation between q value and quantity. We however do observe a correlation between quantity and the c score (which is the used metric to discriminate true from false positive hits in Spectronaut), data completeness and as discussed before number of precursors found (Supplemental Figure 1). We manually compared peak shapes of confidentially identified low abundant precursors and high abundant ones and show a representative example in the new Supplemental Figure 6. There we still see a clear and distinctive signal of only slightly lowered peak shape quality in the low abundant example that can be integrated very well.

Action Taken: We modified Figure 2B and Supplemental Figure 6 accordingly, now showing the number of precursors used for quantification as well. Furthermore, we added the new Supplemental Figure 1 showing the correlation between protein abundance and quality metrics given by Spectronaut as well as the new Supplemental Figure 2 showing the overlap of quantified proteins in single cells vs 0 cell controls and new Supplemental Figure 3 showing exemplary extracted ion chromatograms.

3. Contaminant and zero-cell protein identifications need deeper characterization

The manuscript reports that up to 98% of total detected protein quantity in single-cell samples originates from contaminants, largely because of the very low bacterial protein input and the large excess of trypsin required for sample preparation. This is an important and unavoidable challenge, but it also means that contamination and misassignment must be rigorously addressed.

Given the low CVs observed for apparent bacterial proteins detected in samples that did not contain bacteria, were those bacterial proteins identified in zero-cell controls excluded from all downstream analyses? The authors hypothesize that zero-cell bacterial signals are dominated by noise rather than true peptides, but this should be substantiated with additional metrics.

The authors searched against bacterial proteomes plus the CRAPome contaminant database. However, given the prominent contribution of human-derived contaminants such as keratins, it would be useful to know whether the data were ever searched against a full human proteome database, or a combined bacterial-human-contaminant database, to estimate the extent of human contamination and potential peptide misassignment.

The authors should also clarify whether any bacterial peptides overlap with

human peptides, whether such peptides were removed, and how consistent contaminant protein measurements were compared with bacterial protein measurements.

A direct comparison of CV distributions for bacterial proteins versus contaminant proteins would help determine whether the bacterial measurements behave like true low-input biological signals or more like technical/background signals.

We thank the reviewer for raising this important question and agree that the degree of contamination is much higher in single cell proteomics and especially in single bacteria proteomics compared to bulk level analyses. This is also one of the main reasons why we decided to record 0 cell controls, for being able to check if single cell samples look different from 0 cell control, hence contain real target organism signals. While this is in general the case meaning single cell samples showed more bacterial protein IDs at higher quantity, in line with the reviewers suggestion we made additional checks: We selected the *E.coli* data from figure 2, our evaluation dataset within this study, to check for overlaps of proteins from 0 cell controls and single *E. coli* and found that 10 out of 224 total bacterial proteins found in single cells (~4.5%) were overlapping to negative control samples and this number drops to zero when filtering for a minimum of 3 precursors (Supplemental Figure 2). We did not remove those 10 hits from the single cell pool in our current study and instead show the full results obtained from Spectronaut using default settings. These settings are commonly used for method development/single cell proteomic studies[1–4], with some of them lacking 0 cell negative controls to their data. To increase confidence in our data, we applied the previously discussed minimum 3 precursors used for quantification filter not only to our E coli test-dataset (Figure 2 B) but also to our final Bacillus heat shock sample to focus only on highest confidence hits and automatically exclude proteins overlapping with the 0 cell control (see overlap check in Supplemental Figure 2). We found that the number of non-contaminant proteins identified with the min 3 precursor filter dropped to 9 and 13 (without and with head-shock respectively) but we retain meaningful results in terms of protein regulation and cell clustering (See new Supplemental Figure 7).
We further investigated overlaps to the human proteome as suggested. 1944 proteins (9.4% of the human proteome but 25% of the E.coli proteome) show homology across both species when searching with the NCBI BLASTP (v2.12.0) tool and using an e value threshold of 1E-5, however of those not a single protein pair exceeds 80% sequence identity, and only 16 protein pairs exceed 60% sequence identity. We conducted a search for our single E coli, 0 cell (negative control) and 200 pg *E. coli* (positive control) towards the entire E-coli and human proteome databases and found not a single protein with

cross-species ambiguous annotation. However, we found that a very high number of >1000 human proteins is annotated to the 0 cell and single cells and very high levels of >300 human proteins remain even when applying and additional minimum of 3 precursors and elevated c score cutoff of 5 filter (see Point_to_Point Figure 1). These numbers indicate a high degree of human contamination, maybe caused by human samples previously processed in the same PDC capillary within the cellenONE (despite washing), or introduced by manual handling steps during cell harvest or staining. In line with earlier assumptions, in this data, the CV of presumably real IDs from single E.coli shows a higher protein quantity CV compared to the presumably contaminant human proteins. In our positive control form 200pg bulk, this picture is inversed as here technical replicates are displayed showing even lower CV compared to the random noise contaminants (see Point_to_Point Figure 1a). To ensure that these high number of human matches does not originate from wrongly transferred IDs (despite using method evaluation mode in Spectronaut) or false positive IDs, we started a separate search using the 0 cell negative controls only but found a highly similar result there. When using an Arabidopsis database as entrapment search instead, the number of hits drops dramatically (i.e. from >1200 human proteins found to <60 Arabidopsis proteins found without any additional filters applied, see Point_to_Point Figure 3)

Action Taken: We checked for overlaps in quantified proteins across 0 cell negative controls and single cells (Supplemental Figure 2 added) and found that applying an additional filter for a minimum of 3 precursors used for quantification helps to reduce this overlap from 4.5% to 0% of all proteins. We applied this filter to our final data and still see upregulation of heat shock related proteins in single Bacillus subtilis (Supplemental Figure 7 added). We performed additional searches towards the human proteome as well as entrapment searches to validate the level of human contamination introduced to the samples and FDR level (Point_to_Point Figures 2,3)

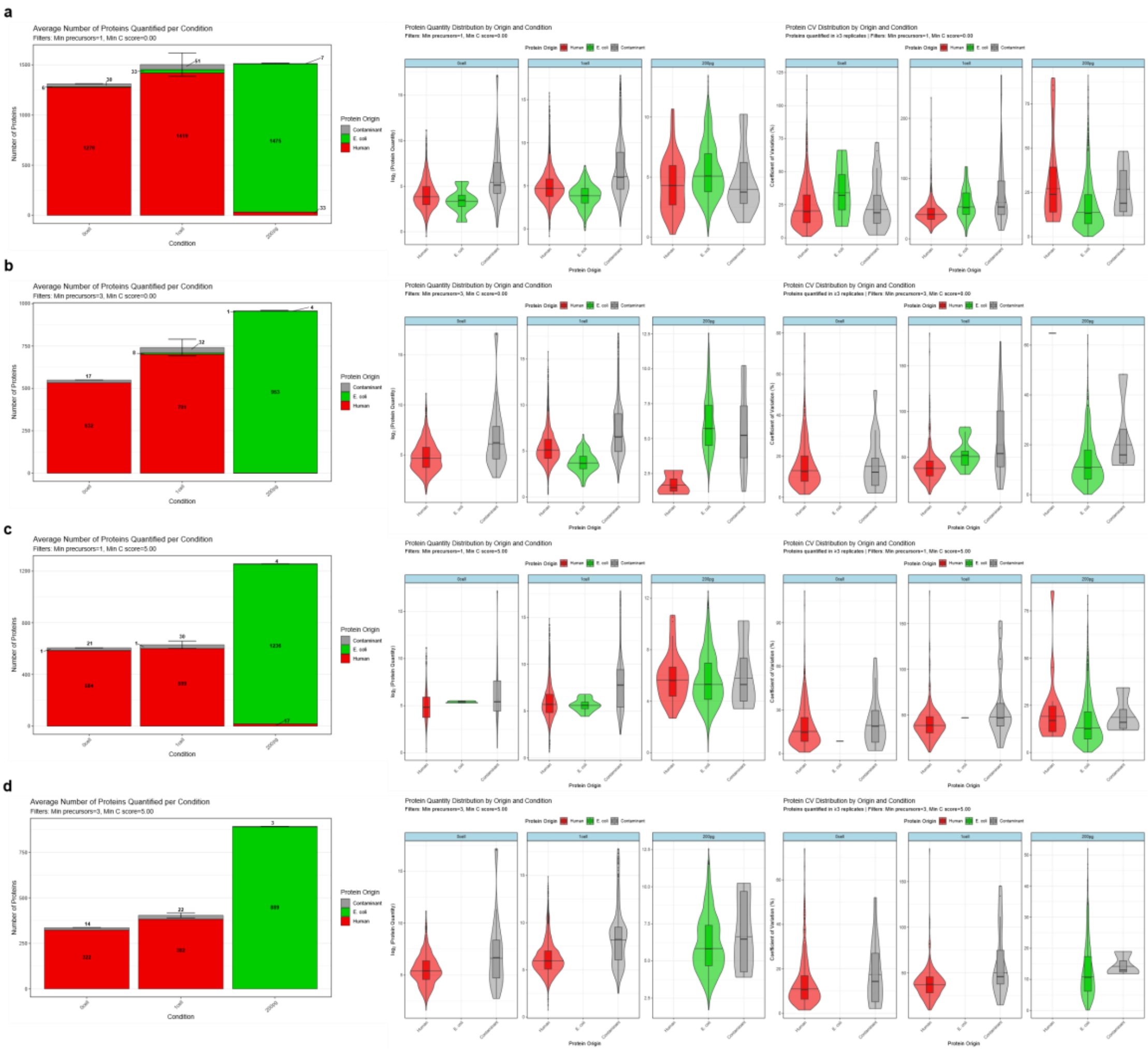

Point_to_Point Figure 2: ***Combined E.coli and human full proteome search***. *Data of Figure 2 from 0 cell controls (n=4), single intact and stained E.coli (n=14) as well as 200 pg E.coli bulk digest (n=3). The plots show (from left to right) the number of quantified proteins, their quantitative distribution and the protein CV when using Spectronaut default filtering options (**a**), or applying an additional minimum of 3 precursors used for quantification (**b**), a minimal c score of 5 (**c**) or a combined filter with a minimum of 3 precursors and a c score of 5 (**d**)*

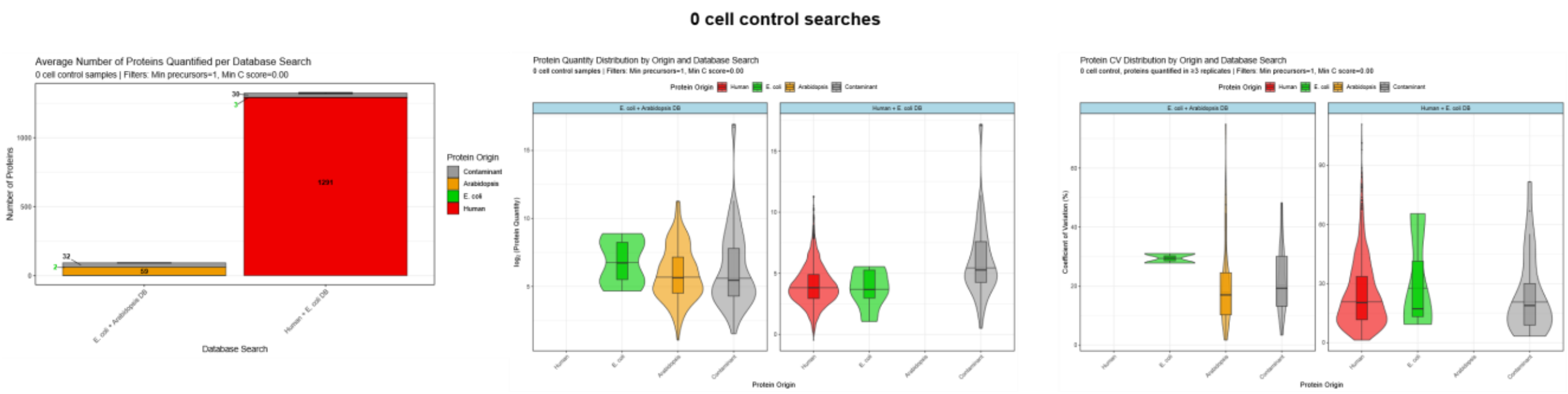

Point_to_Point Figure 3: ***Separate searches of 0 cell control samples towards E.coli and human proteome or towards E. coli and Arabidopsis thaliana proteome as indicated***. *The plots show (from left to right) the number of quantified proteins, their quantitative distribution and the protein CV when using Spectronaut default filtering options. N= 4 samples, Data from Figure 2.*

4. Clarify FDR control and DirectDIA identification strategy at the single-cell level

The data were searched library-free using Spectronaut DirectDIA+, with replicates defined as the same condition, and against species-specific bacterial databases plus CRAPome. Because the manuscript's conclusions rely on very low numbers of detected proteins per cell, it is important to understand how identification transfer, replicate grouping, and FDR control affect the reported single-cell identifications.

The authors should clarify whether identifications were controlled at the run, precursor, peptide, and protein levels independently for each single-cell run, or whether evidence was shared across replicates/conditions. Were zero-cell controls included in the same search?

We apologize for not having a clear enough description in our methods part, which is now corrected. Searches for each figure panel were performed together but in method evaluation mode and with conditions defined to form groups. This means also zero cell controls were included but to the best of our knowledge no matching across conditions should be performed by Spectronaut in method evaluation mode. To doublecheck we performed a separate control search with zero cells only and indeed found minor differences. Searching together yielded 6 bacterial proteins in the 0 cell control (Point_to_Point Figure 2 a) while a separate search yielded only 3 bacterial proteins with exact same search parameters (Point_to Point Figure 3). For figures 2 and 3 in the main manuscript this means that minor changes (likely due to differences in FDR calculation when performing a global vs separate searches) are to be expected if we would search each condition separately.
Regarding FDR control, Spectronauts default settings were used which means a precursor Qvalue cutoff of 0.01 was applied on run and experiment wide level, a precursor PEP cutoff of 0.2, protein Qvalue cutoff of 0.01 and 0.05 at experiment wide and run level respectively, and a PEP cutoff at protein level of 0.75 were applied.

Action Taken: A statement explaining that method evaluation mode was used to avoid evidence sharing across conditions was included. Checks using separate searches and an entrapment search were included (see previous question).

Minor points

1. LC–MS method duration / 80 samples per day
   The authors use an 80 SPD method on the Orbitrap Astral, with a short active gradient. Since sensitivity is the central limitation in this study, it

would be useful to know whether the authors tested slower methods, such as 40–60 SPD, and whether longer gradients or different DIA window schemes improved low-abundance bacterial protein detection. Even a brief statement that this was not tested, or that 80 SPD was chosen to balance depth and throughput, would be helpful.

No other methods have been tested within this specific study, from previous studies[2,5] we know that the sweet spot between coverage and throughput is around 50 SPD for mammalian cells, and from our experience this shifts towards shorter gradients with sharper hence more intense peaks with decreasing input. As a result we decided to go for 80 SPD to balance depth and throughput in this study without further optimization.

Action Taken: We added a statement that 80 SPD was chosen base on previous experience but without dedicating optimization for single bacteria to the methods part of our manuscript as suggested by the reviewer.

2. Definition of “quantified proteins”

The manuscript alternates between “proteins,” “protein groups,” “non-contaminant proteins,” and “quantified proteins.” The authors should define these consistently. For example, are all reported counts protein groups? Are one-peptide protein groups included? Are contaminants excluded from the headline numbers throughout? This is especially important for Figures 2, 3, and 6.

We apologize for the inconsistency within the manuscript and took care to correct this. We are always referring to quantified proteins within this study (never to identified only) and we are always referring to bacterial proteins in headlines and show contaminant numbers separately.

Action Taken: We checked the entire manuscript for inconsistencies to make clear that quantified proteins are meant throughout this entire study.

Textual and grammatical issues

We are grateful to the referee for their attention to detail. All minor issues regarding formatting, typos, and references have been addressed in the revised manuscript.

1. The supplemental figure numbering appears incorrect. The supplement contents list Supplemental Figures 1–3, but the legends label the figures as Supplemental Figures 4–6.
   We corrected this.

2. Around line 132, the text refers to “Figure 1C” when discussing summed protein quantity and diluted bulk comparisons. This appears to refer to Figure 2C, not Figure 1C.
   We corrected this.
3. The text contains “Error! Reference source not found.F” when discussing the PCA of heat-shocked and non-heat-shocked cells. This should likely be corrected to Figure 6F.
   We corrected this broken figure-link and checked for further broken links.
4. “384 well pate” should be “384-well plate.”
   We corrected this.
5. “contaminats” should be “contaminants.”
   We corrected this.

6. “Principial Component Analysis” should be “Principal Component Analysis.”
   We corrected this.

7. Please check spelling and formatting of strain names throughout, including instances such as “B. subitilis” and possible inconsistencies in ΔywlE/ΔywIE notation.
   We corrected this.

Reviewer #3 (Remarks to the Author):

I am generally enthusiastic about this work. Not only is it a monumental achievement to convincingly demonstrate protein measurement from individual bacteria, but the authors made inroads toward showing the utility of the workflow for biological inquiry. Minor comments:

1. The authors should add citations to the first sentence of the introduction
We added current reviews highlighting single cell proteomics by Guo et al (2025), Momenzaded et al (2025) and Hu et al (2026)

2. Line 35, the CellenONE is not the only means of isolating single cells. The authors should also mention and cite works using FACS and Tecan UNO, also perhaps LCM.
We included text and references citing FACS, TECAN and LCM methods to the introduction.

3. Line 85, mention not only active gradients, but samples per day or cycle time.
We added the information that we achieve a throughput of 80 SPD with the 12.5 min active gradient.

4. Figure 2B. Clarify the x axis units. I don't suppose this is copy number? If it were, I don't think it would make sense. Please attempt to convert the protein quantity to

detection limit in terms of copy number. This will be helpful for understanding whether the proteins measured below ~2 on the x axis make sense. We thank the reviewer for this comment. You are correct that the x-axis does not represent the copy number. The x-axis shows the log10-transformed protein group quantities reported as MS2-based area under the curve (AUC) intensities, as output by Spectronaut (SN) v.20 after cross-run normalization. These values represent relative, label-free quantification intensities as commonly used[3,4,6] when acquiring data-independent mass spectrometry data where we cannot infer absolute protein quantities. For quality control, FDR was controlled at the PSM, peptide and protein group level at 1%, with an additional 1% protein q-value cutoff. Quantification of single peptide hit proteins was subject to even more stringent FDR control.

Regarding the limit of detection (LOD) and the limit of quantification (LOQ) in this context: in SN, a protein may be identified (passing FDR control) without receiving a quantitative AUC value when peak areas cannot be reliably extracted, representing the LOD. Proteins that were identified and quantified at the lower end of the x-axis in Figure 2B represent the LOQ. Proteins appearing below ~2 on the x-axis may still represent biologically relevant detections, although they are near the LOQ.

Reviewer #3 (Remarks on code availability):

NA

References Point to Point Reply:

1. Ye, Z. *et al.* One-Tip enables comprehensive proteome coverage in minimal cells and single zygotes. *Nat. Commun.* **15**, 2474 (2024).
2. Bubis, J. A. *et al.* Challenging the Astral mass analyzer to quantify up to 5,300 proteins per single cell at unseen accuracy to uncover cellular heterogeneity. *Nat. Methods* 1–10 (2025) doi:10.1038/s41592-024-02559-1.
3. Krull, K. K., Ali, S. A. & Krijgsveld, J. Enhanced feature matching in single-cell proteomics characterizes IFN-γ response and co-existence of cell states. *Nat. Commun.* **15**, 8262 (2024).
4. Petrosius, V. *et al.* Exploration of cell state heterogeneity using single-cell proteomics through sensitivity-tailored data-independent acquisition. *Nat. Commun.* **14**, 5910 (2023).
5. Hoch, D. G., Belford, M., Heil, L. R., Mechtler, K. & Matzinger, M. Low-resolution FAIMS for increased peptide coverage in low-load and single-cell proteomics. *Sci. Rep.* https://doi.org/10.1038/s41598-026-45228-3 (2026) doi:10.1038/s41598-026-45228-3.
6. Yu, Z. *et al.* Spectronaut and DIA-NN: A Comparison of their Performance in the Analysis of Lung Adenocarcinoma Biopsies. *ACS Omega* **11**, 8080–8093 (2026).
